# DawnRank: discovering personalized driver genes in cancer

**DOI:** 10.1186/s13073-014-0056-8

**Published:** 2014-07-31

**Authors:** Jack P Hou, Jian Ma

**Affiliations:** Department of Bioengineering, University of Illinois at Urbana-Champaign, Urbana, IL USA; Medical Scholars Program, University of Illinois at Urbana-Champaign, Urbana, IL USA; Institute for Genomic Biology, University of Illinois at Urbana-Champaign, Urbana, IL USA

## Abstract

Large-scale cancer genomic studies have revealed that the genetic heterogeneity of the same type of cancer is greater than previously thought. A key question in cancer genomics is the identification of driver genes. Although existing methods have identified many common drivers, it remains challenging to predict personalized drivers to assess rare and even patient-specific mutations. We developed a new algorithm called DawnRank to directly prioritize altered genes on a single patient level. Applications to TCGA datasets demonstrated the effectiveness of our method. We believe DawnRank complements existing driver identification methods and will help us discover personalized causal mutations that would otherwise be obscured by tumor heterogeneity. Source code can be accessed at http://bioen-compbio.bioen.illinois.edu/DawnRank/.

**Electronic supplementary material:**

The online version of this article (doi:10.1186/s13073-014-0056-8) contains supplementary material, which is available to authorized users.

## Background

Recent advances in next-generation sequencing (NGS) technologies have provided us with an unprecedented opportunity to better characterize the molecular signatures of human cancers. The critical challenge facing cancer genomics today is to analyze and integrate such information in the most efficient and meaningful way to advance cancer biology, and then to translate that knowledge to clinic [1,2]. A key question in cancer genomics is how to distinguish ‘driver’ mutations, which contribute to tumorigenesis, from functionally neutral ‘passenger’ mutations [3]. The most basic approach is to categorize mutations based on recurrence, that is, the most commonly occurring mutations are more likely to be drivers [4,5], or by comparing mutation rates in individual genes based on an empirically derived background mutation rate, such as MutSig [6] and MuSiC [7]. Machine learning based approaches use existing knowledge to help identify drivers. For example, CHASM utilizes random forest to classify driver mutations using alterations trained from known cancer-causing somatic missense mutations [8]. There are several recent methods that use additional information to help predict driver genes and driver pathways. CONEXIC was developed to integrate copy number change and gene expression data to identify potential driver genes located in regions that are amplified or deleted in tumors [9]. Network and pathway-based approaches have become one of the most promising methods to understand drivers due to their ability to model gene-gene interactions by aggregating small effect sizes from individual genes. MEMo and Dendrix rely on predicted mutual exclusivity of driver mutations within pathways or subnetworks [10,11]. MEMo utilizes driver cliques based on known pathways with mutually exclusive mutations in the patient cohort, whereas Dendrix identifies subnetworks (*de novo*) with potential driver activity as having high coverage and high mutual exclusivity. Another method, PARADIGM-Shift was developed to utilize pathway-level information along with other features (such as expression, methylation, copy number) to infer gain and loss of function for mutations. PARADIGM-Shift works best with small pathways with specific genes of interest, that is, focus genes [12]. DriverNet classifies driver mutations as mutations that propagate outlying downstream differential expression in the transcriptional regulatory network [13]. More recently, TieDIE [14] was developed to find small cancer driver pathways within a large super-pathway to connect genomic alterations with transcriptomic changes, MAXDRIVER [15] was proposed to identify driver genes by integrating multiple omics data and heterogeneous networks, and VarWalker [16] was developed to construct cancer-specific mutation networks to assist driver identification in a patient cohort. However, the scope of these methods is still limited. Most existing methods such as MEMo, Dendrix, and DriverNet require a large number of patient samples to generate reliable results and lack the ability to discover rare and patient-specific drivers. Other methods such as PARADIGM-Shift are designed to determine drivers in small pathways and often require detailed previous knowledge of specific pathways and focus genes to operate effectively. New methods are needed to identify novel and rare drivers when we do not have much prior knowledge of the tumor.

It is now acknowledged that individual tumors of the same type are highly heterogeneous and have diverse genomic alterations [2,17]. This stems from the ‘long-tail phenomenon’ which states that cancer mutations are characterized by a small number of frequently mutated genes and a large number of infrequently mutated genes [18,19]. Discovering rare drivers in the long tail of genetic alterations remains difficult. Therefore, we urgently need methods to assess the impact of patient-specific and rare mutations from individual tumor samples in order to elucidate personalized molecular drivers.

In this work, we introduce a new method called DawnRank that detects driver genes using data from a single patient sample. By only using data from an individual patient sample rather than a large cohort, we identify drivers in a personalized fashion. The single patient approach classifies drivers regardless of mutation frequency, thereby allowing us to focus on rare (infrequent) drivers. DawnRank ranks potential driver genes based on their impact on the overall differential expression of its downstream genes in the molecular interaction network. Mutated genes with higher rankings are more likely to be drivers. Our method builds on the DriverNet rationale that the impact of a potential driver gene can be determined by its effect on the genes that are regulated by it. However, unlike DriverNet, DawnRank can be applied to a single patient at a time. It is also important to note that our approach differs from the aforementioned PARADIGM-Shift. The small-scale network approach of PARADIGM-Shift works well when the user has a target pathway and genes in mind, but is not applicable in determining rare driver genes distributed across multiple pathways with a more comprehensive gene network. DawnRank differs from MEMo and Dendrix as the scope of DawnRank is to determine individual driver genes rather than driver pathways. Although CONEXIC also utilizes pathway information to identify important CNVs, DawnRank has a more generalized framework to assess the impact of both mutations and CNVs at the individual patient level. Such a patient-specific framework also makes DawnRank different from methods such as MAXDRIVER and VarWalker. DawnRank also differs from TieDIE, which predicts subnetworks of interlinking genes that highlight cancer specific subnetworks, whereas DawnRank predicts individual driver genes.

## Methods

### Method overview

**Figure 1:**
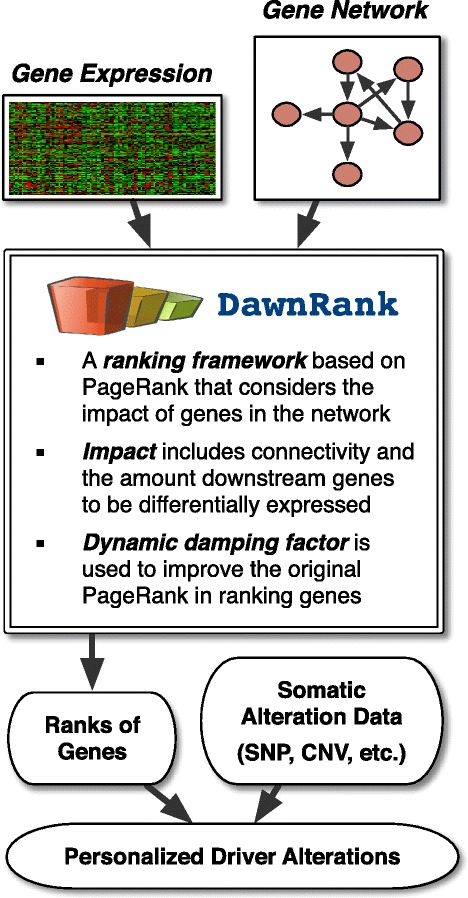
**Overview of the DawnRank method.**

The DawnRank method ranks mutated genes in a single patient according to their potential to be drivers. DawnRank requires the knowledge of a gene interaction network, somatic genomic alterations from the patient’s cancer genome, and the differential gene expression profile between the cancer transcriptome and normal transcriptome. The overview of DawnRank is in Figure 1. Our method ranks genes according to their impact on the perturbation of downstream genes, i.e., a gene will be ranked higher if it causes many downstream genes, directly or indirectly in the interaction network, to be differentially expressed. DawnRank views the gene network as a directed graph. We adopted the random walk approach used in PageRank [20,21] to model this process iteratively. The framework effectively reflects the observation from previous works that mutations in genes with higher connectivity within the gene network are more likely to be impactful. In each iteration, a node in the network can either, with a probability 1 − *d* (*d* is called damping factor, which we defined in a new way. See below.), revert back to stay at the same node, or with a probability *d* to walk randomly to a downstream node, which symbolizes the impact a particular gene has on its downstream neighbors. Our method depends on three parameters: the differential gene expression, the interaction network as a directed graph, and the damping factor. These three parameters along with genomic alterations form the key components of our model to determine drivers in individual patient samples. The output of the rank describes gene’s overall impact. In order to produce a more readable form of the rank, we converted the rank into percentile form to get the relative order of the genes in the rest of this paper.

### The DawnRank algorithm

In DawnRank, a gene will possess a higher impact score (that is, rank) if the gene is highly connected to differentially expressed downstream genes (directly and indirectly connected). Driver genes tend to display a high-degree of connectivity within the gene network [22,23]. For example, using the number of outgoing edges alone, known driver genes as classified by the Cancer Gene Census (CGC) [24] have a mean and median of 31.45 and 12 outgoing edges, respectively, whereas genes not typically classified as drivers (not in CGC) have a mean and median of 17.73 and three outgoing edges, respectively. The higher number of outgoing connectivity of known driver genes suggests that the PageRank model would be appropriate to prioritize driver genes based on their impact in the gene interaction network. PageRank has had several adaptations in genomics. GeneRank utilized PageRank to rank the importance of genes in a molecular network [25]. PageRank derivatives (such as SPIA [26]) have also been used to analyze pathway-level importance. More recently, it was utilized to predict clinical outcome of cancer patients based on gene expression [27] and to assist subtype identification [28]. Such approaches also show similarity in nature to modeling network impact as a heat diffusion process as used in HotNet [29] and TieDIE [14]. DawnRank builds on the original PageRank algorithm by providing a way to model a network’s directionality with more stable rankings by utilizing dynamic damping factors (see below).

DawnRank views the gene network as a directed graph. Let *N* be the number of nodes (in our case, genes) in the directed graph, and *A* be the adjacency matrix representation of the graph, a 0-1 matrix (if node *i* links to *j*, then *A*_*ij*_=1). Note that the current 0-1 adjacency matrix can be naturally extended to consider weighted edges to further distinguish different gene-gene interactions.

We define the rank of each gene iteratively:

1 $$ {r}_j^{t+1}=\left(1-{d}_j\right){f}_j+{d}_j{\displaystyle \sum_{i=1}^N}\frac{A_{j i}{r}_i^t}{ d e{ g}_i},\ 1\le j\le N $$

*r*^*t*^ is the rank in the *t*^*th*^ iteration. The output of the rank describes a gene’s overall impact on the network: the higher the rank, the higher the impact of the gene. *d* is the damping factor, a parameter representing the extent to which the ranking depends on the structure of the graph. In DawnRank, the damping factor is individualized based on gene connectivity (discussed below). *f* is the prior probability of the gene which we set to the absolute differential expression. The absolute differential expression is the absolute value of the difference of the log scale tumor and normal expression values. The $de{g}_i={\displaystyle \sum_{j=1}^N}{A}_{j i}$ is the in-degree of *i*, or the number of incoming edges to *i*. This differs from the original PageRank definition of *deg*_*i*_, which was the out-degree of *i*.

The zero-one gap problem refers to the potential pitfall that assigns biased ranks to some nodes [30]. When trying to rank nodes with 0 incoming edges, known as ‘dangling nodes’, the *deg*_*i*_ will be 0, arising to a divide-by-zero error. In our real gene network data, 15.5% of all genes do not have any incoming edges. The initial PageRank algorithm attempts to handle the problem by setting the damping factor to be 0 for such genes, while using the damping factor 0.85 for all other nodes. If we use this approach, the ranks of genes with no incoming edges will be based solely on its differential expression (and not the network structure). However, this correction in itself causes a large gap in the damping factor for genes with 0 and 1 incoming edge (Additional file 1: Figure S3). This large gap in the damping factor can cause a drastic change in the ranking of the gene when an incoming edge is added to the gene which in turn may cause unstable rankings [30]. An unstable ranking system is especially concerning to gene network data, as it is still not a complete representation of all interactions among genes [31]. Therefore, small modifications and additions to certain gene interactions may significantly alter the rankings of potential drivers. To address this problem, we utilize dynamic damping factors [30], where each gene possesses an individualized damping factor based on the number of incoming edges to that gene (Eq. 2). As the number of incoming edges increases, the damping factor gradually rises to incorporate more connectivity information into the ranking of the gene. Therefore, no large gap is observed from 0 in-degree and 1 in-degree (see Additional file 1: Figure S3).

2 $$ {d}_i = \frac{ d e{ g}_i}{ d e{ g}_i+\mu} $$

The parameter *μ* follows a Dirichlet prior trained from maximizing the values of *μ* over 100 random samples. We selected the *μ* value of 3 because it had the highest average DawnRank scores for known drivers in CGC (see Additional file 1: Supplementary Materials for more details on how *μ* was trained). Overall, the dynamic damping factor mitigates the large change in the damping factor in nodes with 0 and 1 incoming edges by gradually increasing the damping factor as the gene’s in-degree increases, thereby creating more reliable and more stable rankings. A toy example is in the Additional file 1: Figure S2. We also show that DawnRank performs more reliably with a dynamic damping factor than a static damping factor on the TCGA datasets (see Additional file 1: Figure S1).

In addition to the iterative version of DawnRank, the method can also be presented in matrix form:

3 $$ {r}_{t+1}=\left(1- d\right) f+ dM\times {r}_t $$

where *r*_*t*_, *d*, and *f* are *N ×* 1 matrices to represent the rank, gene-specific damping factor, and the gene expression, respectively, and *M* is the transition matrix defined by:

4 $$ \boldsymbol{M} = \left[\begin{array}{ccc}\hfill \frac{A_{1,1}}{de{ g}_1}\hfill & \hfill \cdots \hfill & \hfill \frac{A_{1, n}}{de{ g}_n}\hfill \\ {}\hfill \vdots \hfill & \hfill \ddots \hfill & \hfill \vdots \hfill \\ {}\hfill \frac{A_{n,1}}{de{ g}_1}\hfill & \hfill \cdots \hfill & \hfill \frac{A_{n, n}}{de{ g}_n}\hfill \end{array}\right] $$

DawnRank converges when there is no longer a significant update in the ranks. This is when the magnitude of the difference of the ranks between time *t* + 1 and the previous time point *t* falls below a small *ε*, which we set to 0.001, the same value suggested by [30]. DawnRank also stops when no solution is present after a maximum number of iterations, which we set at 100. In practice, DawnRank always converges for any reasonable *μ* between 0.01 and 20 within 20 iterations. Nonetheless, there are corner cases at low damping factors (μ < 10^− 10^) where DawnRank either does not converge or converges very slowly. A detailed discussion of the convergence of DawnRank can be found in the Additional file 1: Supplementary Materials.

### Condorcet voting for rank aggregation

To aggregate the rankings of genes from individual patient samples to determine the most impactful drivers in a population (for example, known drivers for the same type of cancer or a specific sub-type), DawnRank applies a modified version of the Condorcet method [32]. The Condorcet method is a voting scheme in which ‘voters’ vote for the best ‘candidate’ by submitting a rank-ordered list of candidate preferences. The list of preferences is allowed to be either partial or full. The Condorcet method then selects a winning candidate by comparing every possible pair of candidates *A* and *B* and determining a ‘winner’ by comparing the number of voters that preferred *A* to *B* and *vice versa*. We applied the Condorcet method to the personalized rankings of genes to determine aggregate ranking of genes in a patient population.

Although the Condorcet method is built to handle partial voting lists, one difficulty of implementing the Condorcet method is the lack of patient samples that possess the commonly mutated genes. Many pairwise comparisons are missing for many gene combinations due to the lack of patients that have mutations in both genes simultaneously. However, since DawnRank can output a ranking as an impact score for all genes regardless if a gene is mutated, we evaluated pairwise comparisons of two genes based on patients with a mutation in at least one of the two genes. This approach avoids the use of non-mutated gene comparisons to calculate the aggregate score of genes, as the objective of DawnRank is to determine the altered genes that are the most impactful. However, since mutation recurrence is an important factor in detecting common drivers, we also implemented a penalty heuristic δ, a number between 0 and 1 in our approach to lower the ranking of a gene in a pairwise comparison that is not mutated. This penalty allows us to rank aggregate frequent drivers based on both impact and frequency.

5 $$ \mathrm{PairwiseWinner}\left( A, B\right)=\left\{\begin{array}{c}\hfill A\kern0.5em  if\ \delta (A)\times Rank(A)>\delta (B)\times Rank(B)\hfill \\ {}\hfill\ B\kern7.25em  otherwise\hfill \end{array}\right. $$

where

6 $$ \delta (A)=\left\{\begin{array}{c}\hfill \delta \kern3em \mathrm{if}\ \mathrm{A}\ \mathrm{is}\ \mathrm{NOT}\ \mathrm{mutated}\hfill \\ {}\hfill 1\kern5.25em \mathrm{if}\ \mathrm{A}\ \mathrm{is}\ \mathrm{mutated}\hfill \end{array}\right. $$

We used the output from DawnRank, which we converted to percentile rank format, to represent the ranking of the gene. The penalty heuristic lowers the value of a non-mutated gene when comparing it against a mutated gene. This heuristic serves as both a means to prevent a rare mutation that is impactful in one patient from winning all pairwise comparisons (akin to a candidate winning just because one and only voter that voted for it ranked it higher than any other candidate) and to prevent a low-impact, high-frequency mutation from winning a pairwise comparison against high-impact genes that are not frequently mutated (akin to an unpopular candidate winning just because many voters had a low-preference vote for that candidate). We selected δ by running DawnRank over 100 random patient samples for various instances of δ between 0 and 1 and calculating the precision with respect to CGC genes (see Additional file 1: Supplementary Materials). We found δ to be 0.85.

## Results and discussion

We applied DawnRank to TCGA datasets. We first showed that DawnRank outperforms two recent pathway-based methods DriverNet and PARADIGM-Shift. We also showed that DawnRank produces reliable results as compared to cohort-based approaches CHASM and OncodriveFM. We then used the results of DawnRank to determine both potential novel drivers (new genes mutated frequently), and more importantly, potential rare and personalized drivers that previously could not be assessed by other methods. We also applied the method to study breast cancer subtypes and found that the amount of predicted rare drivers has a strong correlation with the degree of genetic heterogeneity in different breast cancer subtypes.

### Datasets and network

We applied DawnRank to 512 glioblastoma multiforme (GBM) samples, 504 breast cancer (BRCA) samples, and 572 ovarian cancer (OV) samples in TCGA. The datasets we used in this work include gene expression and coding-region mutation data for three cancer types generated by TCGA [33–35]. The data were accessed on 20 May 2013. The mutation data we used included non-synonymous point mutations and insertions and deletions (indels) in coding regions.

We built the gene interaction network used in DawnRank using a variety of sources, including the network used in MEMo [10] as well as the up-to-date curated information from Reactome [36], the NCI-Nature Curated PID [37], and KEGG [38]. The MEMo network consisted of inferred gene-interaction from sources of information such as protein interactions, gene co-expression, protein domain interaction, and text-mined interaction described by [39]. To aggregate all of the networks together, all redundant edges were collapsed to single edges. We included self-loops within the network to account for auto-regulation events [40]. The resulting aggregate network consisted of 11,648 genes and 211,794 edges, and can be downloaded from our supplementary website.

To help evaluate the quality of our results, we obtained a list of 487 known driver genes from the well-studied cancer gene database, CGC [24]. Additionally, we also compared the quality of our results to the Pan-Cancer drivers [41], a driver gene list built using results from well known cohort-based methods over twelve tumor types. In practice, there is no gold standard of known drivers. However, well-curated cancer gene databases such as CGC and the Pan-Cancer study provide an approximate benchmark of known drivers [13,42].

### DawnRank outperforms pathway-based methods DriverNet and PARADIGM-Shift

We evaluated the performance of DawnRank’s ability to identify known drivers and compared it with DriverNet and PARADIGM-Shift. As mentioned above, we utilized CGC as an approximate benchmark of known drivers. Note that here we implicitly assume that all non-synonymous mutations in driver genes are potential driver mutations if they are selected by a method. We performed two separate comparisons: (1) we compared DawnRank to DriverNet over a large network in order to evaluate the performance of the two methods using a large human interaction network (which PARADIGM-Shift is not able to work with practically); (2) we compared DawnRank to PARADIGM-Shift and DriverNet over a smaller, but well-annotated gene network based on KEGG in order to determine the effectiveness of the three algorithms in smaller networks. The network used in the first comparison was the same network described earlier. The network used in the second comparison was a smaller network built from the aggregation of the KEGG cancer pathways with 1,492 gene nodes and 8,070 edges. We ran DriverNet version 1.0.0, defining a differentially expressed gene using their default settings of 2 standard deviations [43], and we ran PARADIGM-Shift version 0.1.9 using the suggested global-rank transformation for expression data [44]. To facilitate the comparison, we applied the Condorcet rank aggregation (see Methods) for the DawnRank scores based on individual patient samples to provide the consensus population-level driver scores (see Additional file 2). For each comparison, we used the following three measures (Precision, Recall, and F1 Score):

7 $$ \begin{array}{c}\hfill Precision=\frac{\left(\#\ \mathrm{Mutated}\ \mathrm{Genes}\ \mathrm{found}\ \mathrm{in}\ \mathrm{CGC}\right)\cap \left(\#\ \mathrm{Genes}\ \mathrm{found}\right)}{\left(\#\ \mathrm{Genes}\ \mathrm{found}\right)}\hfill \\ {}\hfill Recall=\frac{\left(\#\ \mathrm{Mutated}\ \mathrm{Genes}\ \mathrm{found}\ \mathrm{in}\ \mathrm{CGC}\right)\cap \left(\#\ \mathrm{Genes}\ \mathrm{found}\right)}{\left(\#\ \mathrm{Mutated}\ \mathrm{Genes}\ \mathrm{found}\ \mathrm{in}\ \mathrm{CGC}\right)}\hfill \\ {}\hfill {F}_1\  Score=2\times \frac{Precision\times Recall}{Precision+ Recall}\hfill \end{array} $$

**Figure 2:**
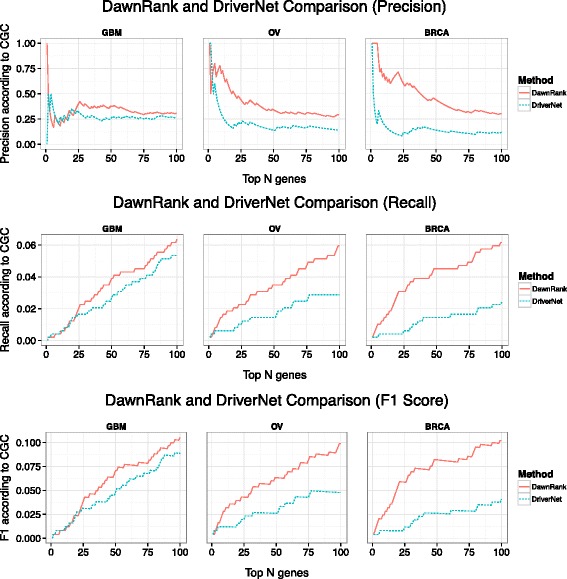
**A comparison of the precision, recall, and F1-scores for the top ranking genes in DawnRank and DriverNet.** The X-axis represents the number of top ranking genes involved in the precision, recall, and F1 score calculation. The Y-axis represents the score of the given metric.

**Figure 3:**
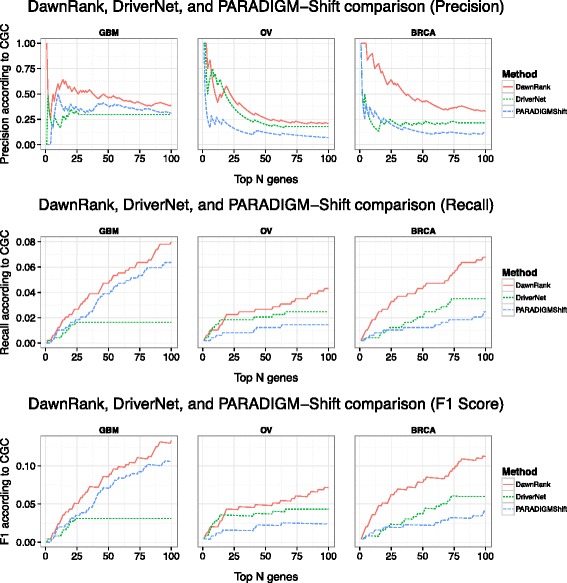
**A comparison of the precision, recall, and F1 scores for the top ranking genes in DawnRank, DriverNet, and PARADIGM-Shift on a small network (defined from the KEGG database).** The X-axis represents the number of top ranking genes involved in the precision, recall, and F1 score calculation. The Y-axis represents the score of the given metric.

Precision, recall, and F1 scores were based on the top *N* genes. We first evaluated the performance between DawnRank and DriverNet. In general, DawnRank outperforms DriverNet in all three cancer datasets with respect to CGC (Figure 2). Although DriverNet performs comparably in ranking the top genes in GBM, it has poorer performance in OV and BRCA. A potential explanation of the difference may lie in the total number of mutations in the three cancer datasets. GBM had 5,478 mutations over 599 genes, while OV had 13,520 mutations over 4,968 genes and BRCA had 11,900 mutations over 5,205 genes. The numbers indicate that there may be more passenger mutations in BRCA and OV and DawnRank is less affected by noise than DriverNet. An illustration of this is DriverNet’s ranking of the gene *TTN* as a top 5 driver in both BRCA and OV. *TTN* is the longest gene in the human genome and recent TCGA analysis has suggested that that higher mutation rate in *TTN* is likely to be artifacts [34]. *TTN* was not ranked among the top 60 genes in any cancer according to DawnRank. We then evaluated the performance of DawnRank, PARADIGM-Shift, and DriverNet using the smaller KEGG network. Overall, DawnRank outperforms both DriverNet and PARADIGM-Shift in terms of precision, recall, and F1 scores using CGC as a standard (Figure 3) or the Pan-Cancer results as a standard (Additional file 1: Figure S11). In BRCA, although some known drivers such as *TP53* and *ATM* were detected by multiple methods, DawnRank detected important known driver genes in the top 10 such as *CDH1* and *PIK3R1*, and *BRCA1* in breast cancer which were not detected by either PARADIGM-Shift or DriverNet as top ranking drivers.

**Figure 4:**
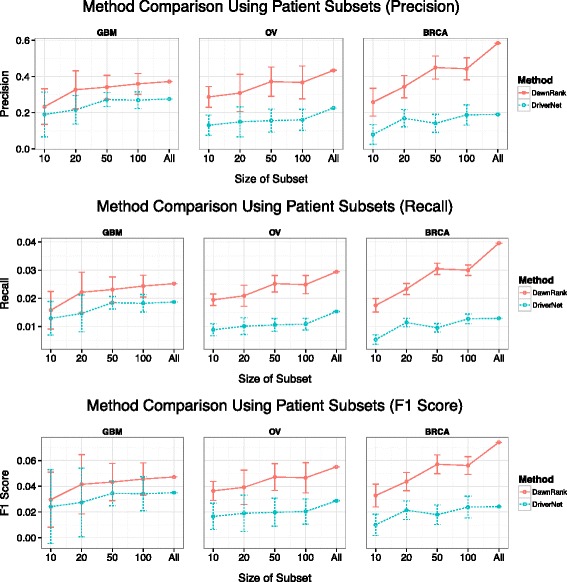
**Comparison results using subset of patient samples.** The figure shows the precision, recall, and F1 scores of the DawnRank and DriverNet results in determining known drivers among their top 30 genes using a small subset of the patient samples (X-axis) rather than the entire cohort. The Y-axis represents the average precision of 10 runs using the subset. The error bars show the 1-standard deviation range of each data point.

In addition to its ability to more precisely identify known driver genes at the entire patient population level, DawnRank also has an advantage of obtaining high-quality results from a much smaller patient cohort. To evaluate this ability, we applied DawnRank to random subsets of the patient cohort from GBM, OV, and BRCA from 10, 20, 50, and 100 patients to determine the precision at which DawnRank identifies known drivers in its top 30 results. We compared these results with that of DriverNet using the same patient subsets. The mean of the precision scores after 10 runs is presented in Figure 4. We found that even with a small patient cohort, DawnRank can still perform reasonably well, much better than DriverNet. Even though DawnRank does not perform as well with a sample of 10 as it can with the entire dataset, the average DawnRank precision at 10 samples is comparable to the DriverNet precision using the entire cohort. Our results suggest that DawnRank is able to identify known drivers even with a small number of samples.

### DawnRank achieves reliable results as compared to cohort-based approaches CHASM and OncodriveFM

In addition to comparing DawnRank to the pathway-based approaches of DriverNet and PARADIGM-Shift, we compared DawnRank to non-pathway-based approaches as well. We compared DawnRank with CHASM [8] and OncodriveFM [45]. We ran CHASM version 3.0 using CRAVAT [46] with the cancer driver analysis mode [47], and we ran OncodriveFM using the IntOGen Mutation Analysis interface using SIFT, Polyphen2, and Mutation Assessor data [48]. Both CHASM and OncodriveFM can rank genes from most likely drivers to least likely ones. Like in DawnRank, we evaluated all non-synonymous mutations and evaluated each method using precision-recall metrics in comparison to CGC. The results (Additional file 1: Figure S10) show that DawnRank performs comparably to both CHASM and OncodriveFM. These results also demonstrate that the personalized approach of DawnRank can achieve reliable results at the cohort level.

### DawnRank can discover both novel and personalized drivers

In this section, we use DawnRank to identify driver genes that may not have been classified as drivers by other methods. We discuss such driver genes determined by DawnRank in two main categories: general novel drivers, and personalized drivers (we are mostly interested in personalized rare drivers).

- A general novel driver is a recurrent driver that has not been classified as a driver gene. Here we define novel drivers as genes that satisfy the following requirements: (1) Highly ranked (genes that have an aggregate rank in the top 30 of driver genes based on the patient cohort), which means it is considered as driver in multiple samples; (2) Recurrent (alteration frequency >2% in the patient cohort); (3) Not previously classified as a driver by CGC.
- A personalized driver is a gene predicted to be a driver for specific patients. Here we define personalized drivers as drivers classified as significant using DawnRank’s maximally selected rank statistics cutoff (explained later) and as drivers that display higher than expected rankings under the Chauvenet’s criterion for detecting outliers. In other words, personalized driver genes could be recurrently mutated in multiple samples, but they are only ranked high enough to be considered as drivers in specific samples. We are particularly interested in rare drivers, as rare drivers would be obscured by the long tail phenomenon of cancer mutations. Personalized drivers mutated in less than 2% of patients will be considered personalized rare drivers.

#### Discovering novel drivers with coding mutations

Using the criteria listed above to detect novel drivers, we used the aggregate DawnRank score to select genes with coding mutations that were considered novel drivers. In GBM, we found several consistently high-ranking potential novel driver genes. Of them, *TGFBR2, HIF1A,* and *FOXO3* are the most promising. These genes are highly ranked, frequently mutated, and are involved in several cancer functions and pathways (see Additional file 3). *TGFBR2* is a transforming growth factor whose function is to regulate cell division signals and inhibit cell division [49]. *TGFBR2* is the third-ranked driver in terms of Condorcet aggregate score, and it is altered in 18.7% of GBM patient samples. *TGFBR2* is classified as a potential tumor suppressor (it shows decreased expression in GBM samples), and it is involved in many pathways to cancer including TGF*β* signaling, HTLV infection, and Hippo signaling [50]. *HIF1A* is a hypoxia inducing factor that has been shown to be necessary for the survival of cancer stem cells in gliomas [51]. *HIF1A* is involved in mTOR pathway, which regulates nutrient sensing for cell growth, which could be responsible for cell growth and metastasis in cancer. *HIF1A* is ranked as the fourth most impactful driver in GBM, and it is mutated in 13.4% of GBM patient samples [52]. *FOXO3* is the eighth-ranked gene*.* It is a common phosphorylation target of *AKT* and *ERK,* and is a key trigger for apoptosis in the *PIK3/AKT* pathway [53].

In OV and BRCA, there were fewer candidate novel drivers than GBM, however, we found two genes that show strong potential to become novel drivers: *PDPK1* in OV and *CENPE* in BRCA. *PDPK1* is a phosphoinositide dependent protein kinase-1 that interacts with a crucial driver gene *AKT1*, and together their signaling plays a critical role in activating proliferation and survival pathways within cancer cells such as the *PIK3/AKT* pathway and the mTOR pathway [54,55]. *PDPK1* is ranked second in OV and is mutated in 5.4% of OV samples. The mutation in *PDPK1* itself also suggests an important impact in the functionality of the protein binding. For example, in sample TCGA-13-0751, the mutation is in a Glycine to Arginine substitution near a hairpin loop in the middle of the protein (Additional file 1: Figure S6). The amino acid change from G to R changes the dynamic of the protein structure by replacing a non-polar amino acid with a positively charged amino acid, which may change the binding interaction with the negatively charged substrate phosphatidylinositol-3,4,5-trisphosphate, which in turn may affect the phosphorylation and activation of at least 24 kinases [56].

In BRCA, a potential novel driver we found is *CENPE. CENPE* is a centromere-associated protein that contributes to mechanics of microtubule-chromosome interactions with mitotic checkpoint. Overexpression of *CENPE* can lead to excessive cell growth and contribute to tumor proliferation [57]. *CENPE* is ranked 26th in DawnRank. We observed that it is highly overexpressed in tumor samples*. CENPE* is interesting as it is ranked differently among different subtypes of breast cancer. All but one of the mutations in *CENPE* was in Luminal B or Basal subtype. The Basal subtype mutations had the highest average ranking in the 95.2 percentile and the average Luminal B subtype mutations ranked lower in the 90.9 percentile, whereas the Luminal A mutation was much less significant with a ranking in the 78.3 percentile. This shows that even though *CENPE* has mutations in multiple subtypes, it may harbor different driver potentials in different subtypes.

#### Discovering novel drivers with copy number changes

We then applied DawnRank to examine the role of CNVs in the three cancer types used in this study. We included genes that displayed at least a two-fold change in copy number change (with respect to the normal baseline) and also had a corresponding change in expression. We treated CNV events in a similar fashion as coding mutations to prioritize the genes with an amplification and deletion and then determined the most impactful CNVs. In addition to known driver genes with CNVs, we also identified novel driver CNVs not in CGC (Additional file 1: Figure S7).

In GBM, known CNV drivers (based on CGC) include the amplification of some well-known driver genes such as *EGFR*, *PDGFRA*, and *MYC* and the deletions of *MLLT3* and *ANK3*. A potential novel driver CNV is the amplification of *SEC61G. SEC61G* is a proto-oncogene that is required for tumor cell survival [58]. *SEC61G* is altered in 14.3% of GBM cases and it is ranked 17th among CNVs. *ELAVL2* is a potential tumor suppressor gene whose function is to stabilize and control nervous-specific binding proteins [59]. *ELAVL2* is altered in 6.7% of GBM cases and is ranked second among CNVs in GBM. In OV, many known drivers are amplifications such as *CCNE1* and *KRAS* and deletions such as *MAF* or *PIK3R1*. An example of a novel amplification is *FZD5*, a ‘frizzled’ gene that has a strong co-expression event with the Wnt signaling pathway in ovarian cancer [60]. *FZD5* is altered in 2.9% of OV cases and it is ranked ninth among CNVs in OV. In BRCA, known driver CNVs include amplifications in *CCND1, MYC, GATA3,* and *EGFR* and deletions in *MAF*. An example of a novel amplification event in BRCA is *PAK1,* which is a breast cancer oncogene that activates *MAPK* and *MET* signaling pathways [61]. *PAK1* is the third ranked gene with CNVs, and it is altered in 2.4% of the BRCA patient samples.

#### Discovering personalized drivers

In this section, we discuss the personalized scope of DawnRank by demonstrating its ability to determine personalized novel and rare drivers. The main aspect that distinguishes DawnRank from existing programs is the ability to discover rare and even patient-specific drivers. Even if a gene is altered only in a single patient, DawnRank will still be able to evaluate the impact of that alteration. We determined such personalized drivers using the ‘maximally rank statistics’ method. This method classifies alterations as ‘drivers’ and ‘non-drivers’ by assigning a cutoff that maximizes the number of known driver genes (genes in CGC) ranking higher than the cutoff). Altered genes in the individual patient with a ranking higher than the cutoff would be described as personalized drivers. The average cutoff for GBM, OV, and BRCA was 98.1, 92.8, and 92.2, respectively (measured in percentile rank). We specifically looked for genes that have not been classified as a driver in CGC and genes that have a significantly higher than expected rank in specific patients. A rank is considered significantly high in a patient if the rank is considered as an outlier under Chauvenet’s criterion for outlier detection.

**Figure 5:**
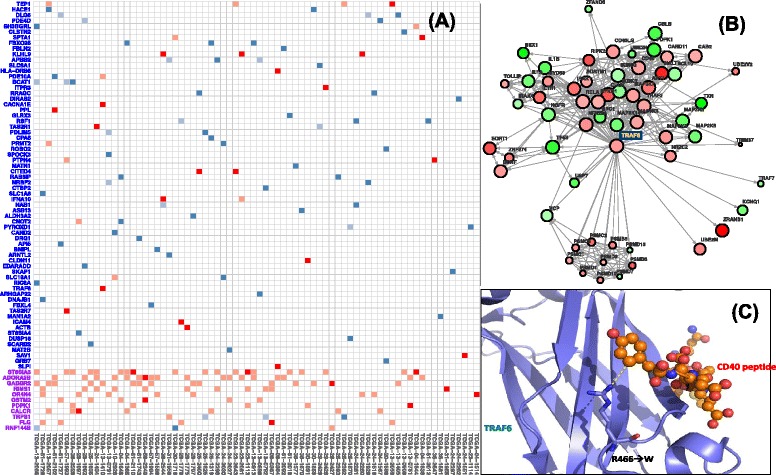
**Personalized drivers in TCGA ovarian cancer samples. (A)** The darker red/blue entries (red for point mutations and blue for CNVs) indicate the personalized drivers that are significant and not documented in CGC. The lighter entries are mutations that are not considered as drivers by DawnRank in specific samples. The X-axis includes patient samples with a personalized driver that is significant and not documented in CGC. On the Y-axis, rare drivers (frequency <2%) are in blue and non-rare drivers (frequency >=2%) are in purple. **(B)** Part of the gene interaction network where *TRAF6* is involved. The size of the node scales according to the DawnRank score in ovarian cancer patient sample TCGA-13-1410 and the color intensity scales with differential expression. Note that this figure shows the impact of *TRAF6* to other genes in the network, but not all the edges where *TRAF6* is not involved are shown due to space limitation (for example, the large number of outgoing edges of *TP53*). **(C)** A close-up view of the protein structure of *TRAF6* indicates that an amino acid change from the mutation in ovarian cancer patient sample TCGA-13-1410 causes substitution of the Arginine (R) to Tryptophan (W). The substitution of the mutation occurs in a conserved binding site, and the substitution from a charged Arginine to a non-polar aromatic amino acid Tryptophan may suggest a change in the substrate binding with the CD40 peptide substrate.

In our case, a gene is considered to be a rare driver if the gene is both classified as a driver using the above criteria, and it is mutated in only a small number of patients (<2%). We selected genes that fit the above criteria to discover potential personalized drivers: genes that labeled as significant from the maximally-selected rank statistics and genes that ranked higher in specific patients according to the Chauvenet’s criterion. These selection criteria yielded in 26 potential personalized drivers in GBM (10 from mutations, 16 from CNV), 56 potential personalized drivers in BRCA (20 from mutations, 36 from CNV), and 77 potential personalized drivers in OV (26 from mutations, 51 from CNV) (Figure 5A and Additional file 1: Figures S8 and S9).

We found 26 potential personalized drivers in GBM. Of these genes, we found that several of the candidate driver genes were involved in important known cancer pathways. Using KEGG to map genes to pathways (see Additional file 3) [50], mutations in three interferon receptor genes *IFNA14, IFNW1,* and *IFNA17* belong to multiple pathways that have significant impact on cancer, including the Cytokine-Cytokine receptor pathway and the JAK/STAT pathway. Cytokines are important factors in tumor cell control as they are key players in inflammatory and immune response [62]. JAK/STAT is a related pathway activated by the binding of cytokines to its receptor. The pathway is involved in inflammation, proliferation, and invasion/migration [63]. In either case, *IFNA14, IFNW1,* and *IFNA17* are interferon receptor genes that may interact with genes such as *TP53* to induce apoptosis of cancer cells [64]. Both the Cytokine-Cytokine receptor pathway and the JAK/STAT pathway are common drug targets in GBM [62,63], which could lead to the implications that *IFNA14, IFNW1,* and *IFNA17* could be targeted. *IFNA14, IFNW1,* and *IFNA17* are mutated in 4.7%, 5.6%, and 4.5% of GBM patients, respectively. *IFNA14* is ranked significantly higher than its average expected ranking in patients TCGA-32-1978 and TCGA-19-2619 while *IFNW1* and *IFNA17* both have high-ranking mutations in the patient TCGA-28-1749. Although *IFNA14, IFNW1,* and *IFNA17* are examples of personalized drivers, they are not personalized rare drivers. An example of a personalized rare driver is *PITX2*, which is altered in only two patients (0.4%). *PITX2* is in the TGF*β* signaling pathway, which can be targeted by cancer drugs such as decanoyl-RVKR chloromethylketone in GBM [65,66].

We found 77 potential personalized driver genes in OV, and like GBM, many of these genes fall under similar KEGG pathways (see Additional file 3). Figure 5A shows the list of personalized drivers in OV. The most well represented KEGG pathways are the calcium signaling pathway and the MAPK signaling pathway. Calcium signaling is important in the regulation of cancer cell proliferation and/or apoptosis [67]. In OV, four alterations are present in the calcium signaling pathway: mutations in *ADORA2B, CACNA1E,* and *ITPR3* along with a CNV for *SLC8A1*. Of these alterations, *ADORA2B* is often associated with cancer cell movement and metastasis, and it has been a drug target in other cancers [68]. The MAPK signaling pathway plays a role in communication with cell growth, and it can be targeted by cancer drugs such as *MEK* and *RAF* inhibitors [69,70]. Genes mutated in the MAPK signaling pathway include *CACNA1E, TRAF6*, and *DUSP16*. The *TRAF6* gene is especially interesting as it upregulates *HIF1A* and has implications in tumor angiogenesis [71]. *TRAF6* is another good example to demonstrate the power of DawnRank because it is mutated in only two patients, TCGA-13-1410 and TCGA-61-1919, but DawnRank was still able to detect it as a rare driver. We looked into the *TRAF6* mutation further, including its connectivity in the network and the potential functional impact of the mutation (Figure 5B and C). We found that the mutation in TCGA-13-1410 is considered to have an important functional impact by MutationAssessor, affecting the MATH domain [72]. The mutation is located at a loop defining one wall of the binding site and projects its long side-chain to directly contact the conserved aromatic residue in the peptide substrate, and it likely modulates its interaction with its receptors [73]. *TRAF6* is also known to be involved in the NF-κB pathway, which is related to poor outcomes in ovarian cancer [74].

We found 56 potential rare drivers in BRCA. Unlike in GBM and OV, the distribution of rare drivers in BRCA seemed to spread out among different pathway with no major cancer pathway having more than two candidate rare drivers. Nevertheless, several important cancer pathways are highlighted in BRCA (see Additional file 3). One of them is the KEGG ‘Proteoglycans in Cancer Pathway’. Proteoglycans bind to ligands and receptors that regulate tumor neoplastic growth and angiogenesis [75]. Alterations in genes coding for proteoglycans include *ANK3* and *FRS2*. Of these, *FRS2* is especially interesting due to both its scarcity (only one amplification event in all BRCA patients) and its function as a fibroblast growth factor whose amplification activates the *FGFR2* [76]. It is also highly differentially expressed in the patient, having differential expression 3.21 standard deviations above normal. The calcium signaling pathway, like in ovarian cancer, also has multiple potential drivers in breast cancer. These genes include *CACNA1B* and *GRIN2A. GRIN2A* is a potential rare driver as it is mutated in 1.7% of BRCA patients, but it has been known to be recurrent driver mutation in other cancers [77].

#### Distribution of personalized rare drivers across breast cancer subtypes

**Figure 6:**
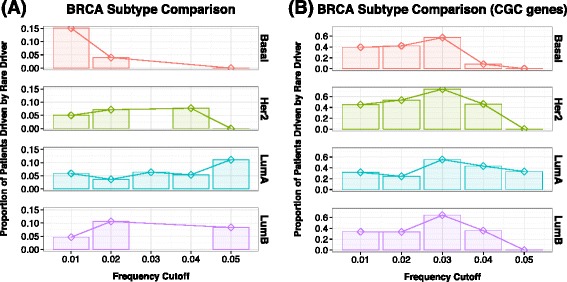
**Distribution of rare drivers (A) and known drivers (B) across four breast cancer subtypes.** The X-axis describes the frequency cutoffs (1% to 5%). Note that this cutoff is not cumulative. The Y-axis shows the proportion of patient samples that have rare drivers within that frequency cutoff.

We next looked at the distribution of the candidate rare drivers across breast cancer subtypes. We tested whether or not the rare drivers show different distribution among patients in the four major subtypes of breast cancer (Luminal A, Luminal B, Her2, and Basal-like). Although we used a 2% as a threshold to determine rare drivers so far, we further modeled the personalized drivers at low frequencies using cutoffs from 1% to 5% to better visualize the distribution of infrequent, personalized drivers in BRCA subtypes. As shown in Figure 6A, the distribution of the driver genes is not the same among subtypes. The most obvious contrast involves the Basal-like and Luminal A comparison. Basal-like samples tend to have more very rare drivers occurring in less than 1% of the cases and have much small number of drivers occur over 1%. Luminal A samples, on the other hand, have fewer drivers occurring in less than 1% of the samples, but as the cutoff increases, more Luminal A drivers can be found. A Chi-squared goodness of fit test confirms that the distribution of Basal and Luminal A breast cancers have very different distributions (*P*=0.015). This may suggest a fundamental difference in the genetic heterogeneity of Basal-like and Luminal A breast cancers. The rare driver distribution of Basal-like cancer is also different from the frequency distribution of known driver genes (Figure 6).

The distribution of known drivers and rare drivers may correspond to the genetic heterogeneity of breast cancer subtypes. Luminal A is an ER+ subtype with less heterogeneity and has been defined by its good prognosis. Patients with Luminal A subtype generally have the most favorable outcome and have a smaller chance of relapse when treated early [78,79]. On the other hand, the Basal-like subtype, which mainly comprises of triple negative breast cancers, is difficult to treat due to tumor heterogeneity. Basal-like breast cancers are known to be more aggressive with poor prognosis [35,80]. Based on our results, we found that Luminal A samples seem to have common drivers more than any other cancer type. The pattern in the Basal-like type is the opposite. There are more potential rare drivers in Basal-like samples than other subtype. This suggests that Luminal A breast cancers are driven more by common drivers while Basal-like breast cancers are driven more by rare drivers.

Potential personalized rare driver alterations for Basal-like breast cancer include *LGR5* and *BCL2L14. LGR5* is a CNV that is amplified and overexpressed in the Basal-like patient sample TCGA-AN-A0AT. *LGR5* is necessary for the stimulation of basal cell growth in breast tissue [81]. *BCL2L14* is a key regulator of cell apoptosis at the mitochondria level [82], and it is altered in the single Basal-like patient TCGA-AN-A0FJ.

### Implementation and running time

DawnRank was implemented in R. Using a network of 1,492 nodes and 8,070 edges and a cohort of the BRCA samples we used, we compared the run time of DawnRank, DriverNet, and PARADIGM-Shift on a computer with 16GB RAM and Intel i5-3317U processor. DriverNet was the fastest, with a runtime of 6 min. DawnRank completed the analysis in 9 min for the main script and 12 min for the Condorcet rank aggregator. PARADIGM-Shift took approximately 15 h to run.

## Conclusions

It is now acknowledged that individual tumors of the same type are highly heterogeneous and have diverse genomic alterations. Therefore, we urgently need novel methods to identify patient-specific and rare drivers from individual tumor samples in order to elucidate personalized molecular mechanisms in different types of cancer. The goal of DawnRank is to integrate mutation data, gene expression, and network information to discover drivers in a personalized manner. We applied DawnRank to a large number of TCGA datasets. By comparing to previous studies, our results demonstrated the effectiveness of DawnRank: (1) Despite its single-patient scope, DawnRank detects common and known drivers with as much or more precision than existing methods; (2) We can identify rare and novel genes that are potential drivers to specific patients. We believe this method will complement existing driver identification methods and will help us discover potential personalized drivers. The application to breast cancer subtypes further demonstrated that the rare drivers predicted by DawnRank may provide new insights into the molecular explanations of cancer subtypes with higher tumor heterogeneity.

One potential limitation of the current DawnRank method is that we still largely rely on known molecular interaction network. Such network information is still incomplete, which may create false negatives in DawnRank’s prediction. In addition, currently the overall network information we use in DawnRank is not cancer-type specific or patient-specific. Therefore, some of the network level interactions and perturbations specific to certain cancer subtypes or patient samples may be obscured by this approach. Another potential limitation is that DawnRank detects only potential drivers that alter the expression of other genes. However, this may not be the case for all drivers. In addition, some of the predictions from our method could potentially be caused by passenger mutations that coincidentally change the expression of downstream genes. In those cases, it may be useful to additionally utilize cohort and functional-impact based methods such as OncodriveFM. As the community continues to refine our understanding of the interaction networks and cancer driver genes, we expect that DawnRank’s ability to predict driver alterations would also improve.

Our method provides a unique solution to predict potential driver genes in cancer. However, the computationally predicted personalized drivers should not be over-interpreted before additional experimental evidence becomes available. To fully validate such computational predictions, both *in vitro* and *in vivo* experimental validations would be needed to comprehensively assess the tumorigenic potentials of predicted drivers in individual patients. Nevertheless, our method provides a reliable solution to prioritize such follow-up experimental validations. Taken together, our personalized approach is promising to discover potential causal generic variants that would be otherwise obscured by tumor heterogeneity. The personalized framework may help determine the optimal treatment strategy for each patient through individualized assessment based on the molecular signatures of their cancers.

## Additional files

Additional file 1 **Supplementary materials.**

Additional file 2 **Aggregate ranking of genes in GBM, OV, and BRCA.**

Additional file 3 **Pathway information for known drivers and personalized drivers.**

## Abbreviations

BRCA: Breast Cancer
CGC: Cancer Gene Census
GBM: Glioblastoma multiforme
NGS: Next-generation Sequencing
OV: Ovarian Cancer
TCGA: The Cancer Genome Atlas
