# Supplementary material for: DawnRank: discovering personalized driver genes in cancer
Source: Genome Med. 2014 Jul 31;6(7):56. doi: 10.1186/s13073-014-0056-8 (PMC4148527; doi:10.1186/s13073-014-0056-8)
Supplement: Supplementary file 1 — Supplementary materials. [file 13073_2014_56_MOESM1_ESM.pdf]

DawnRank: Discovering Personalized Driver Genes in Cancer (Supplementary Materials)

Jack P. Hou and Jian Ma

Comparison between the dynamic damping factor and the static damping factor

We compared DawnRank using the dynamic damping factor (see Methods section) to a PageRank-like algorithm with a static damping factor to determine the impact of our method over traditional PageRank-like methods. Precision, recall, and F1 score were based on the top *N* genes where *N* ranges from 1 to 100. The PageRank-like static damping factor used the PageRank-suggested value 0.85, whereas the DawnRank dynamic damping factor used a free parameter trained μ of 3 (see Methods in the main text). We evaluated the precision and recall scores to compare the quality of the static damping factor and the dynamic damping factor.

In evaluating both damping factors, the dynamic damping factor has a higher precision, recall, and F1 score than the static damping factor in all three cancers. See Supplementary Figure S1. In OV and BRCA, many known drivers such as *NF1, BRCA2, RB1,* and *APC* in OV and *PIK3CA, MAP2K4,* and *PIK3R1* in BRCA were ranked in the top 10 using the dynamic damping factor, and they were not accounted for using the static damping factor. In GBM, the dynamic damping factor performs slightly worse regarding the top 10 genes as several driver genes such as *TP53* and *EGFR* are ranked in the top 15 rather than the top 10.

Overall, the result shows that the dynamic damping factor is helpful in determining known driver mutations more precisely than the static damping factor.

The Zero-One Gap Problem

An illustration of the zero-one gap problem is shown in Supplementary Figure S2. In Supplementary Figure S2, there is a network with six nodes linking to a center node. Each of the outer nodes linking to the center node has the same ranking. In the PageRank-like method with a static damping factor, the rank of a node depends on the rank of outgoing nodes, and therefore genes that have more outgoing edges would have a higher score. However, when static damping factor is used, adding outgoing edges from the central node provides a counterintuitive result, not increasing the rank of the central node while also decreasing the ranks of the outer nodes. This is due to the difference in the damping factor between a node with no incoming edge and a node with one incoming edge is very large (a difference of a damping factor 0 and 0.85). This tends to lead to unstable rankings. The DawnRank dynamic damping factor correctly stabilizes the rank of the outer

nodes while augmenting the rank of the central node. The change in rankings demonstrates the importance of using a dynamic damping factor. As shown earlier, we also compared the results on using dynamic damping factor and static damping factor based on TCGA data, and demonstrated that the dynamic damping factor can help identify more drivers than the static damping factor (Supplementary Figure S1).

DawnRank Proof of Convergence

The DawnRank formula is shown in Equation 1 below:

$$r_j^t = (1 - d_j)f_j + d_j \sum_{i=1}^{N} \frac{A_{ji} r_i^{t-1}}{deg_i}, 1 \leq j \leq N \tag{1}$$

To prove that the convergence time is small, let us define r_j^* to be the true ranking of any gene j. Therefore the true rank of any gene must satisfy the DawnRank formula in (1) exactly.

$$r_j^* = (1 - d_j)f_j + d_j \sum_{i=1}^{N} \frac{A_{ji} r_i^*}{deg_i}, 1 \leq j \leq N \tag{2}$$

To determine the convergence, we calculate the error of DawnRank at each time point t compared to the true result. The error rate of gene j will be defined as the sum of the absolute difference between each ranking of gene j:

$$Err(t) = \sum_j |r_j^t - r_j^*| \tag{3}$$

To calculate the error, we calculate $r_j^t - r_j^*$:

$$r_j^t - r_j^* = \left(d_j \sum_{i=1}^{N} \frac{A_{ji} r_i^{t-1}}{deg_i} - d_j \sum_{i=1}^{N} \frac{A_{ji} r_i^*}{deg_i} \right) \tag{4}$$

Therefore, by way of the triangle inequality $|r_j^t - r_j^*|$:

$$|r_j^t - r_j^*| \leq d_j \left(\sum_{i=1}^{N} \frac{A_{ji} |r_i^{t-1} - r_i^*|}{deg_i} \right) \tag{5}$$

And the error rate will become the summation for all j.

$$Err(t) = \sum_j |r_j^t - r_j^*| \leq \sum_j d_j \left(\sum_{i=1}^{N} \frac{A_{ji} |r_i^{t-1} - r_i^*|}{deg_i} \right) \tag{6}$$

This becomes

$$Err(t) \leq \sum_{i=1}^{N} \frac{\sum_j (d_j A_{ji}) \, |r_i^{t-1} - r_i^*|}{deg_i} \tag{7}$$

Since $deg_{\mathrm{i}} = \sum_{j=1}^{N} A_{ji}$ and $0 \leq d_j \leq 1$ for all instances of j:

$$\frac{\sum_j (d_j A_{ji})}{\sum_j (A_{ji})} \leq 1 \tag{8}$$

We have

$$Err(t) \leq \sum_{i=1}^{N} \frac{\sum_j (d_j A_{ji}) \left| r_i^{t-1} - r_i^* \right|}{\sum_j (A_{ji})} \leq Err(t-1) \tag{9}$$

Unless the damping factor for each j is equal to 1, the error will decrease at a compound rate. Therefore given enough iterations, the $Err(t)$ will eventually approach 0. In our damping factor: $d_i = deg_i/(deg_i + \mu)$, where $\mu = 3$, the damping factor will always be less than 1 for all genes. Therefore, for our iteration of DawnRank, the algorithm will converge at a compound rate.

Parameter Training

We trained two parameters in our model, the μ free parameter used to calculate the damping factor $d_i = deg_i/(deg_i + \mu)$, and the δ parameter used in the Condorcet rank-aggregation in order to account for missing data in certain pairwise comparisons. We performed the parameter training over 100 random patient samples using the small network of the KEGG pathways of 1,492 genes. This network was used to avoid using the same data to train the model and to run the analysis. This model was run over a 10-fold cross validation to determine the most reliable results.

Calculating μ. We calculated μ by running the DawnRank algorithm using the data and network described in the main text over various empirically chosen μ. We selected the μ that presented the highest average rank for common driver genes from CGC. Our results show that the μ parameter has a peak at μ=3 where the average percentile rank of known driver genes is in the 72.7 percentile. Known drivers are driver genes in CGC. In Supplementary Figure S4, we show that μ=3 parameter contains the highest average rank of common driver genes with downward trends as μ both increases and decreases, so we set μ=3. DawnRank scores are based on both differential expression and a network connectivity, whose weight is determined by the damping factor. A high μ value lowers the impact of the network in DawnRank, and a low μ puts too much emphasis on the network and not enough on the differential expression.

Calculating δ. We then applied the Condorcet rank aggregation to our DawnRank trial runs using the μ=3. Since the δ is a value from 0 and 1, we ran the Condorcet rank aggregation for all values of δ from 0 and 1 with an increment of 0.05. We selected the parameter that maximized the precision with respect to genes in CGC for the top 10 mutated genes within the randomly selected patient samples. This process was run 10 times, and the precision results for the 10 runs are shown in the Supplementary Figure S5 below. We can see from the figure that the precision with respect to CGC is maximized at $\delta = 0.85$.

Comparison against summary statistics

In addition to comparing DawnRank results with well-established and known methods, we also compared DawnRank rankings to simple metrics. We compared DawnRank's rankings to the rankings based on the results: (1) if we only used connectivity of the genes; (2) if we only used differential expression of the genes; and (3) if we only used the maximum downstream differential expression, i.e., ranking genes by the maximum differential expression among all outgoing genes. We used the same precision-recall evaluation metrics. The results showed that none of the three metrics performs well by themselves, suggesting that it is necessary for DawnRank to use a combination of connectivity and differential expression to calculate its rank (Supplementary Figure S12).

Comparison against tumor quality and survival rate

We examined potential clinical factors that may show relationships with the DawnRank output. We compared the DawnRank scores with tumor quality and survival rate. With tumor quality, we found no significant correlation with DawnRank scores. For each patient in the three cancer types, we determined the Pearson correlation of the tumor nuclei percentage with both the average rank of driver mutations and the variance of the ranks of the driver mutations. This would test whether or not the tumor nuclei percentage was correlated with either the rank of driver mutations, indicating that more tumor quality leads to more significant driver, or the variance of the driver mutations, indicating that tumor quality raises the variation among drivers. The resulting correlation, however, was low (each of the correlations for driver means and variances with tumor nuclei percentages was between 0.01 and -0.01) with the tumor quality having little relationship with the mean or variance of drivers. One potential reason behind this lack of correlation is the fact that TCGA has already required tumors should have at least 60% tumor nuclei for data from NGS platforms (and 80% for previous tumor samples).

We then examined the relationship between the DawnRank scores and the survival rate. We took advantage of the inter-tumor heterogeneity of the tumors, and the resulting DawnRank scores were used as variables to build a consensus hierarchical co-clustering model using Wards linkage to separate the patients into potential subtypes. We compared our results to the gene-expression based GBM subtypes: Classical, Proneural, Neural, and Mesenchymal [1]. We chose to analyze GBM because it has both gene expression based subtypes and lower and more variable survival rates [2]. The results are shown in Supplementary Figure S13. Using a log-rank test, we found that the DawnRank hierarchical clustering separated the subtypes by survival time even more accurately than that of conventional gene-expression subtypes ($p=0.0044$ and $p=0.131$).

Relationship between DawnRank drivers and the mutation rate

One factor that may determine the number of driver mutations predicted by DawnRank could be the mutation rate itself. On average, there are 4.24 drivers per patient in GBM, 5.89 drivers in BRCA, and 6.05 drivers in OV, within the range of the 2-8 mutations expected in solid tumors [3]. The total number of alterations (mutation plus copy number) in each of the three cancers is 7,876, 10,950, and 17,189, respectively. Therefore, even though OV has higher mutation rate, the number of drivers only increases a little, suggesting the robustness of our method. To examine the DawnRank score in a more extreme case, we applied our method to the lung cancer data in TCGA, including 22,789 mutations across 152 samples. We found that the average number of drivers in lung cancer patients is 7.18. Therefore, even with the large number of mutations in lung cancer, DawnRank still predicts the reasonable number of drivers, further suggesting the robustness of the method. We also plotted the number of predicted drivers against the total number of mutations for individual patients in Supplementary Figure S14. We observed that as the number of mutations increases, the number of predicted drivers in an individual sample increases at a much lower rate until it reaches a plateau, further suggesting the robustness of our method.

Supplementary Figures

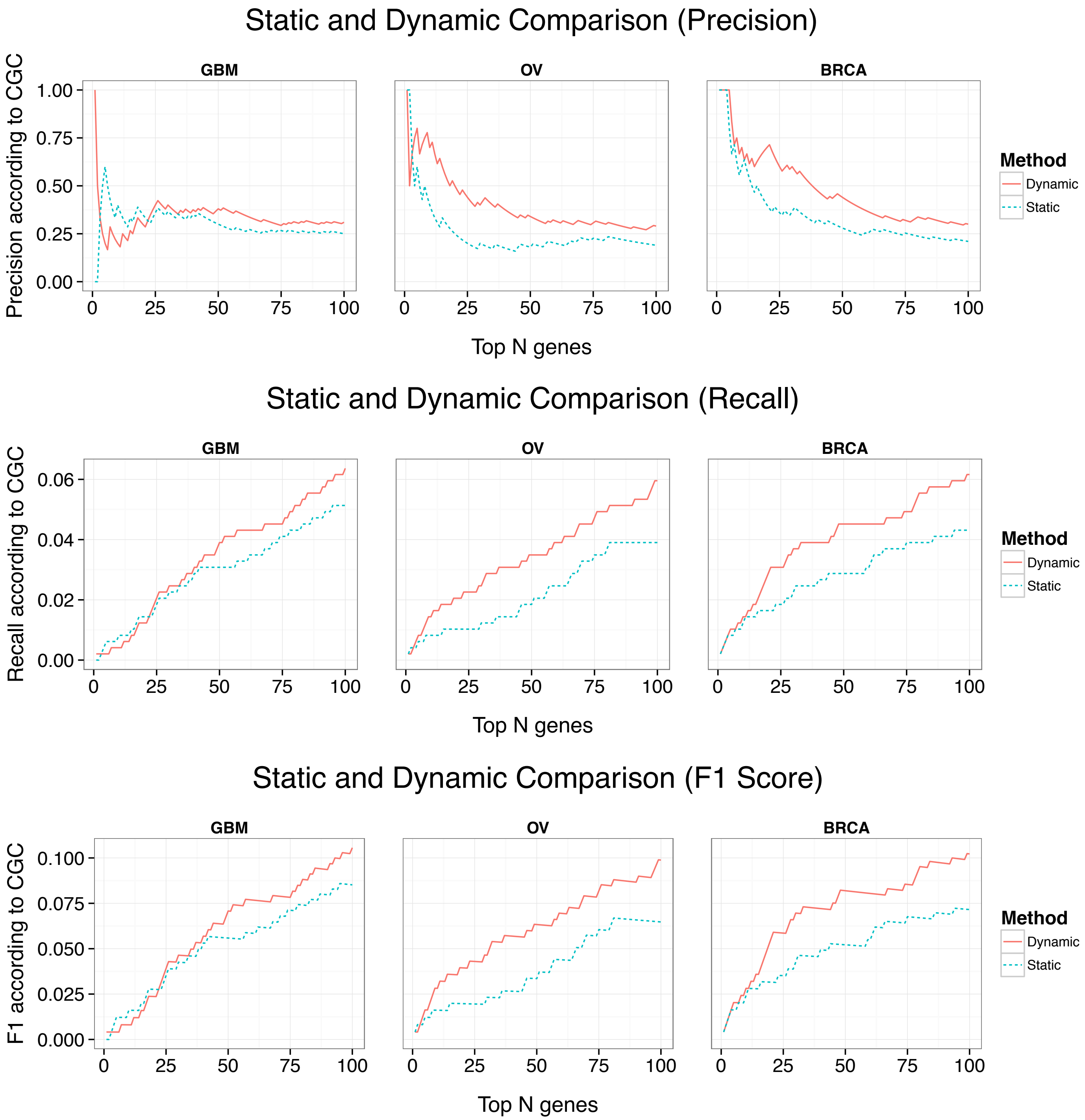

Figure S1: A comparison of the precision, recall, and F1 score for the top ranked genes when using the static damping factor (in original PageRank) and the dynamic damping factor (in DawnRank). The X-axis represents the number of top ranked genes involved in the precision, recall and F1 score calculation, and the Y-axis represents the score of the given metric.

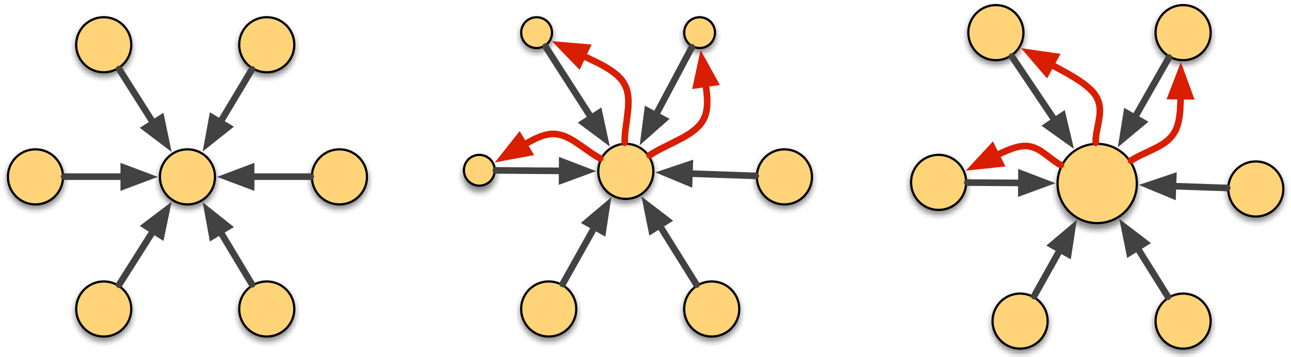

(A) **(B)** **(C)**

Figure S2: A toy example showing the effect of using dynamic damping factor. **(A)** Original network with one central node and six outer nodes. **(B)** When static damping factor is used, after adding the outgoing edges (in red) from the central node, the ranking of the central node does not change and the ranking of the outer nodes decreases. **(C)** When dynamic damping factor is used (as in DawnRank), after adding the outgoing edges from the central node, the ranking of the central node increases (as expected) and the ranking of the outer nodes does not change (as expected).

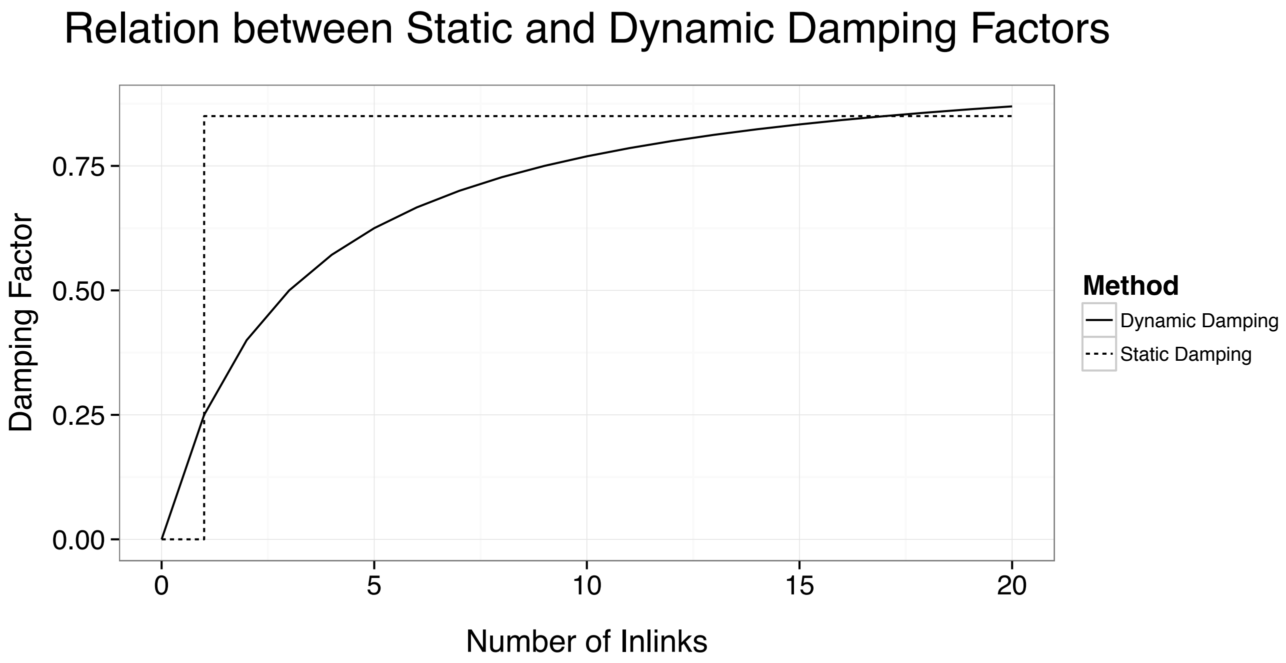

Figure S3: The static versus the dynamic damping factor change over the number of inlinks (i.e. incoming edges).

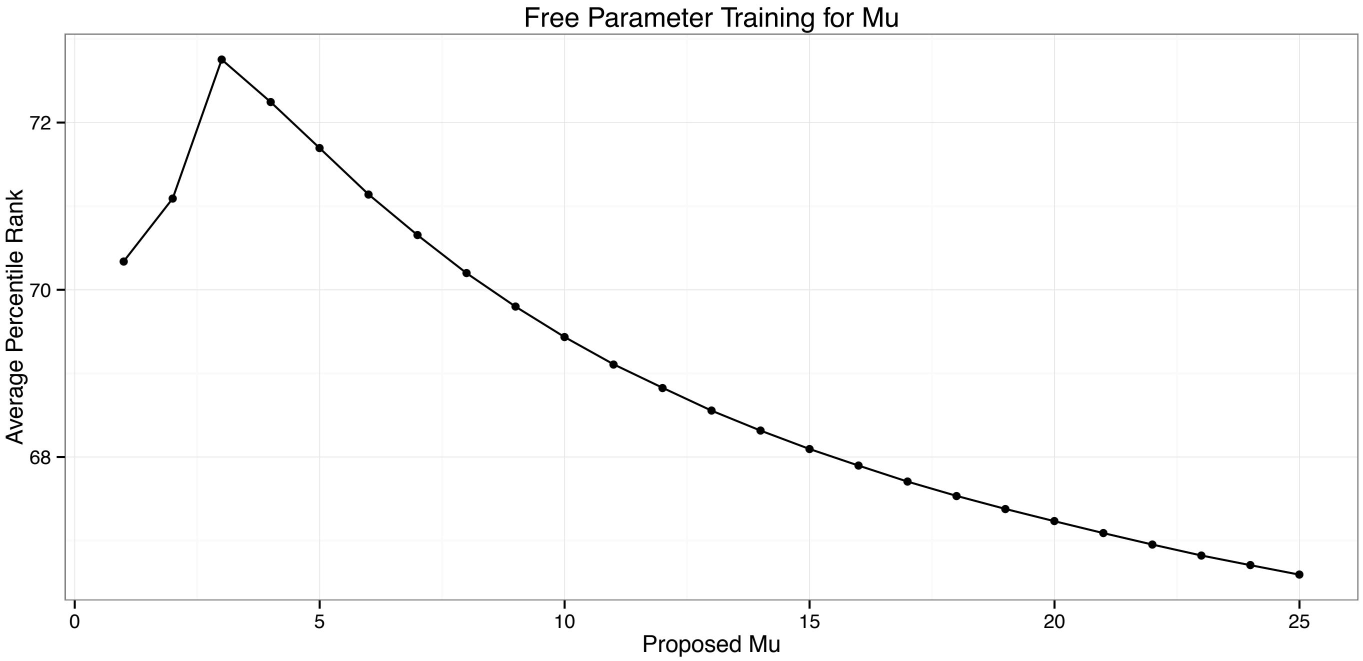

Figure S4: The training results for various μ parameters when looking at the average rank of common driver genes. The μ that provides the highest average rank is 3.

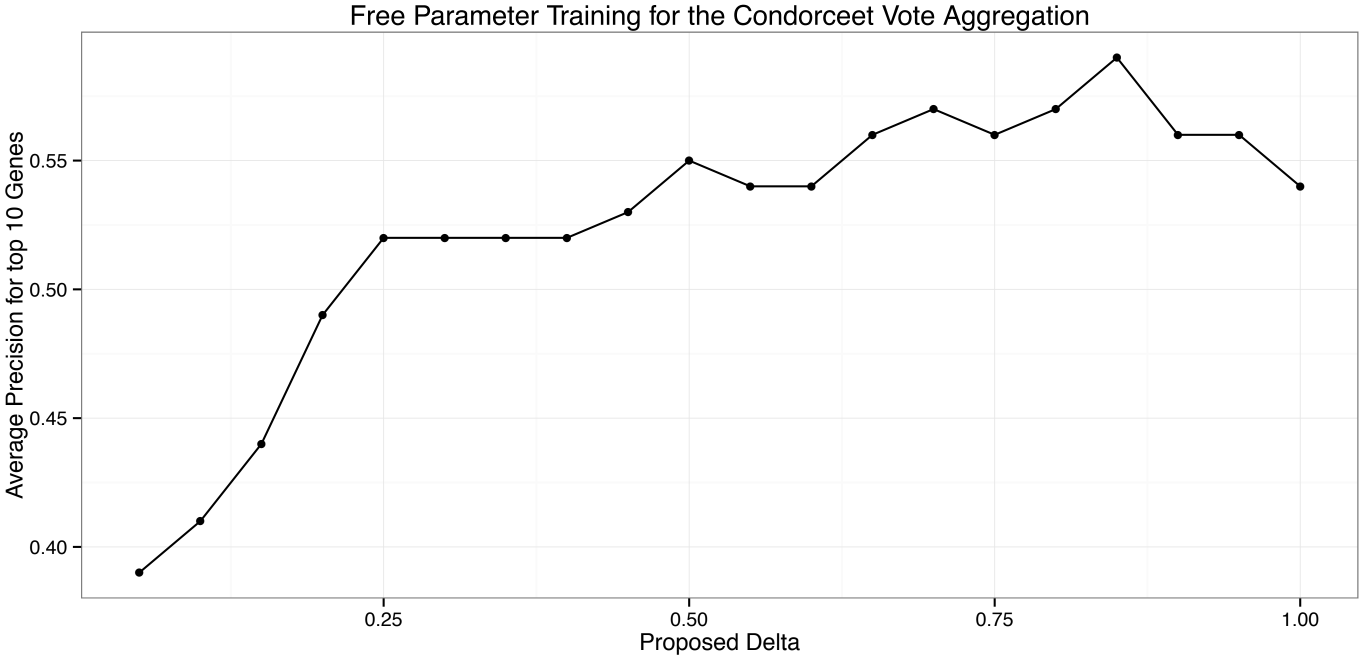

Figure S5 The training results for various δ parameters when looking at the average precision of the top 10 rank-aggregated genes with respect to CGC. The δ is maximized at 0.85 with a precision of 0.59.

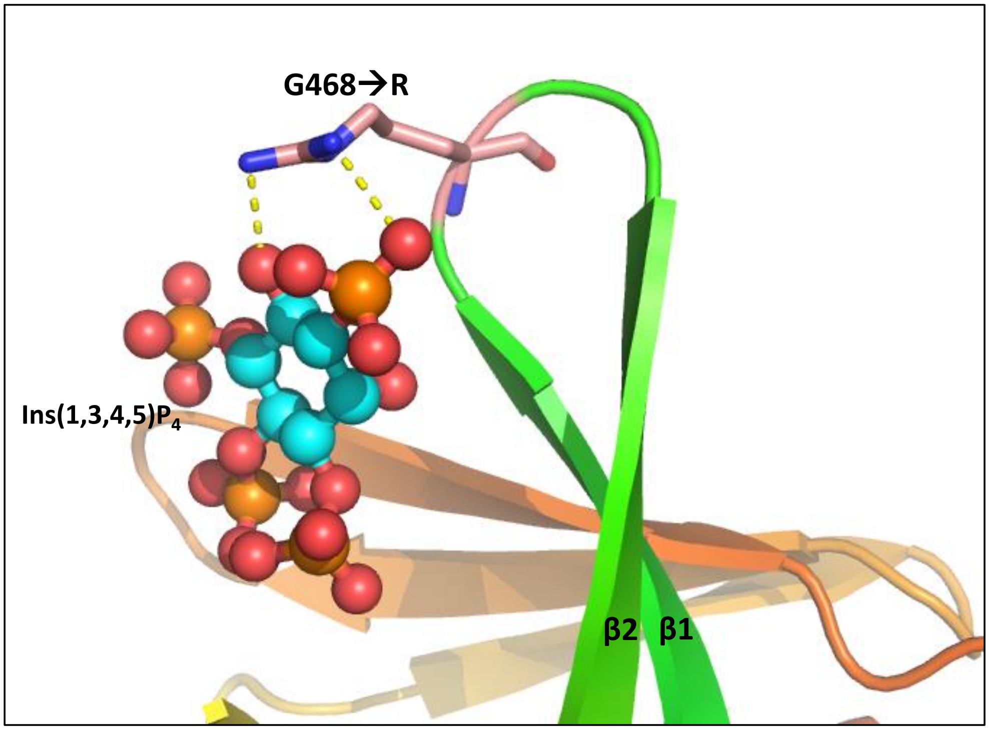

Figure S6: A close-up view of the protein structure of PDPK1 indicates that an amino acid change from the mutation causes substitution of the Glycine (G) to Arginine (R) in TCGA Ovarian Cancer samples TCGA-13-0751 (DawnRank score 98.14 percentile). The substitution is in a loop between 2 beta strands, and occurs close to the binding site for the substrate Ins(1,3,4,5)P4, indicating a potential interaction of the positively charged R-group of Arginine and a phosphate group.

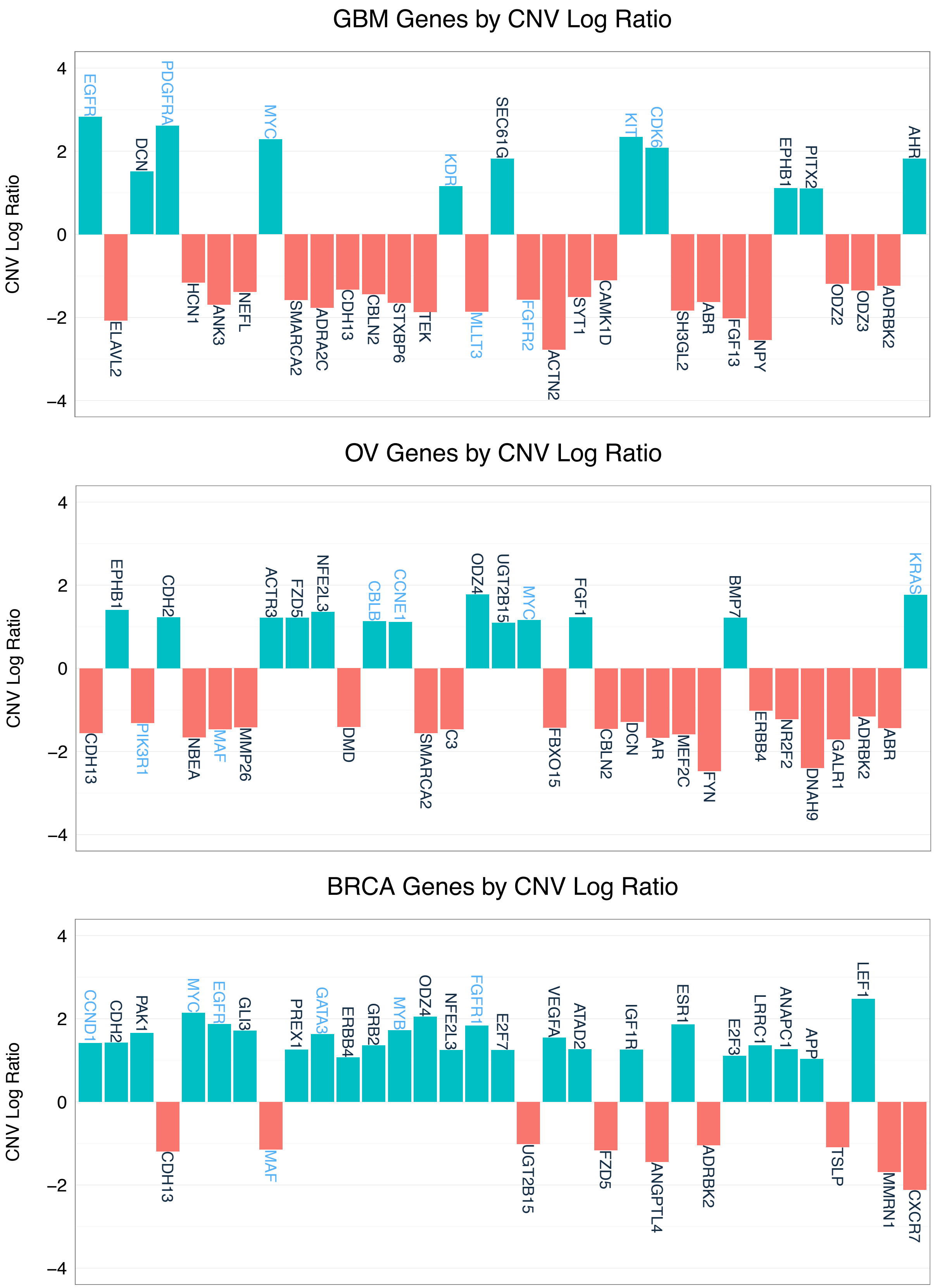

Figure S7: A visualization of the top driver CNVs in BRCA, GBM, and OV. The X-axis represents the top genes (in order of rank), and the Y-axis represents the copy number changes. The labels of known drivers are shown in blue.

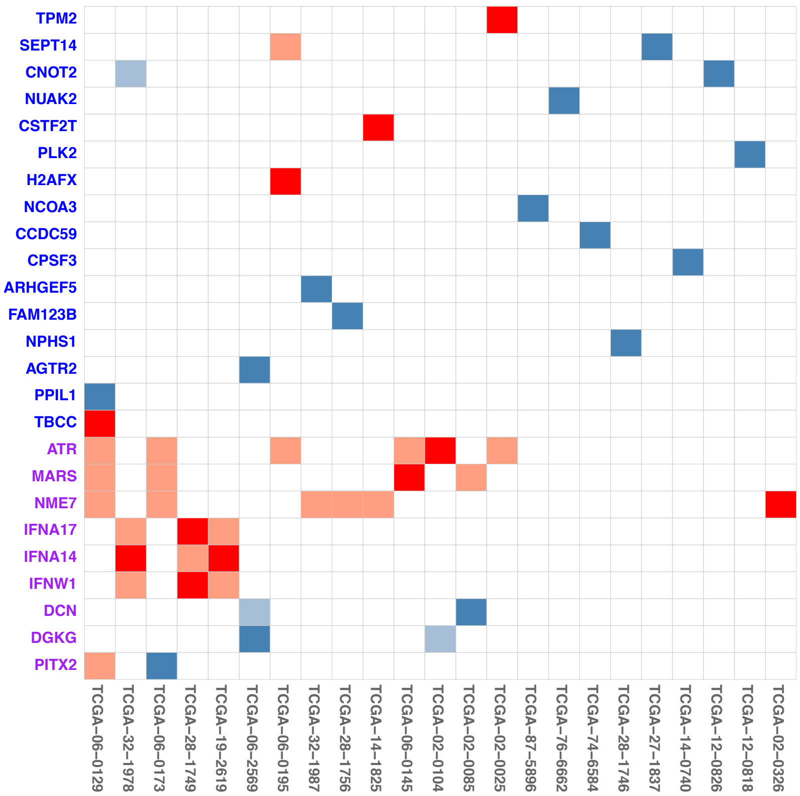

Figure S8: Personalized drivers in TCGA GBM samples. The darker red/blue entries (red for point mutations and blue for CNVs) indicate the personalized drivers that are significant and not documented in CGC. The lighter entries are mutations that are not considered as drivers by DawnRank in specific samples. The X-axis includes patient samples with a personalized driver that is significant and not documented in CGC. On the Y-axis, rare drivers (frequency < 2%) are in blue and non-rare drivers (frequency >= 2%) are in purple.

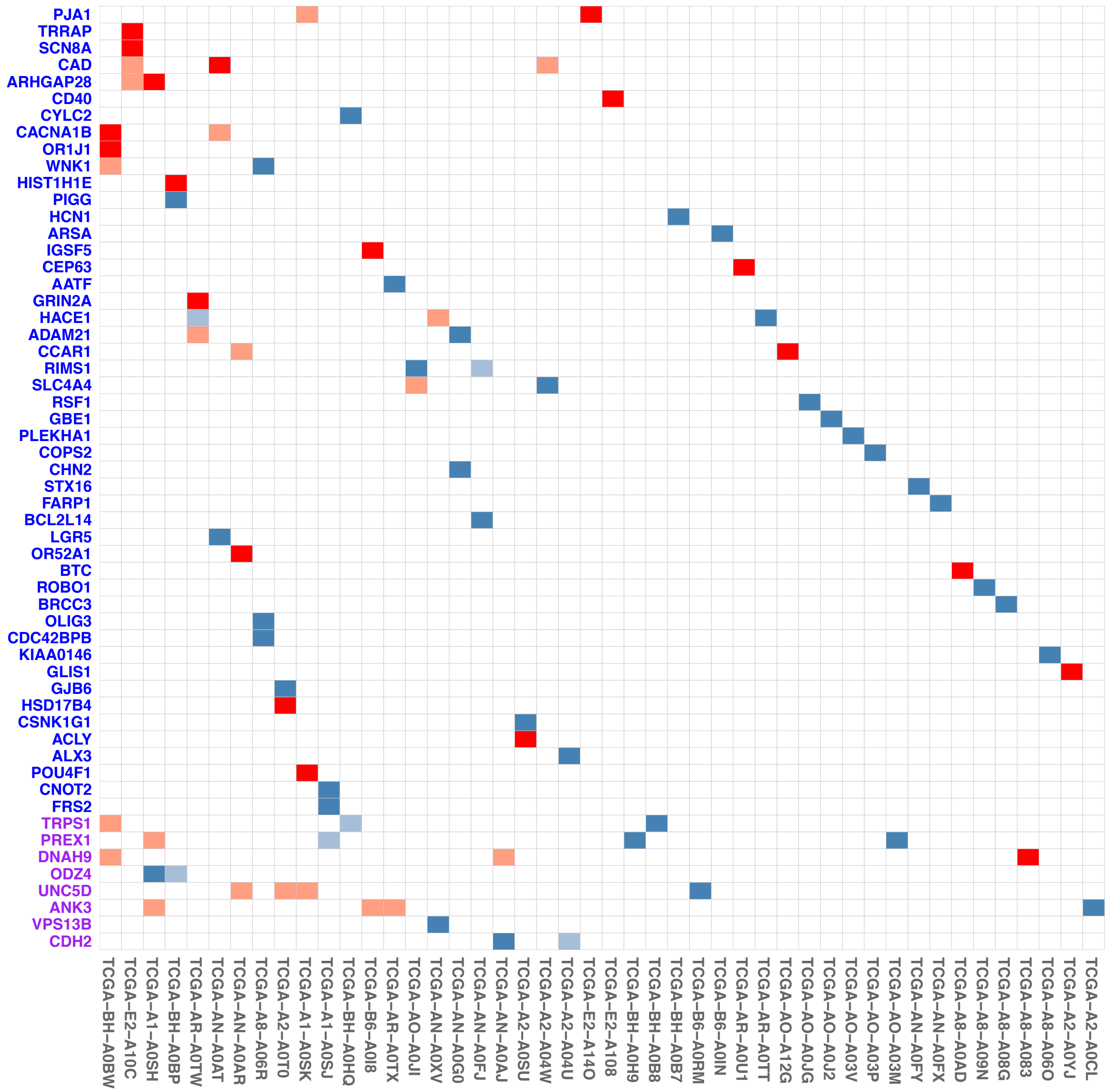

Figure S9: Personalized drivers in TCGA BRCA samples. The darker red/blue entries (red for point mutations and blue for CNVs) indicate the personalized drivers that are significant and not documented in CGC. The lighter entries are mutations that are not considered as drivers by DawnRank in specific samples. The X-axis includes patient samples with a personalized driver that is significant and not documented in CGC. On the Y-axis, rare drivers (frequency < 2%) are in blue and non-rare drivers (frequency >= 2%) are in purple.

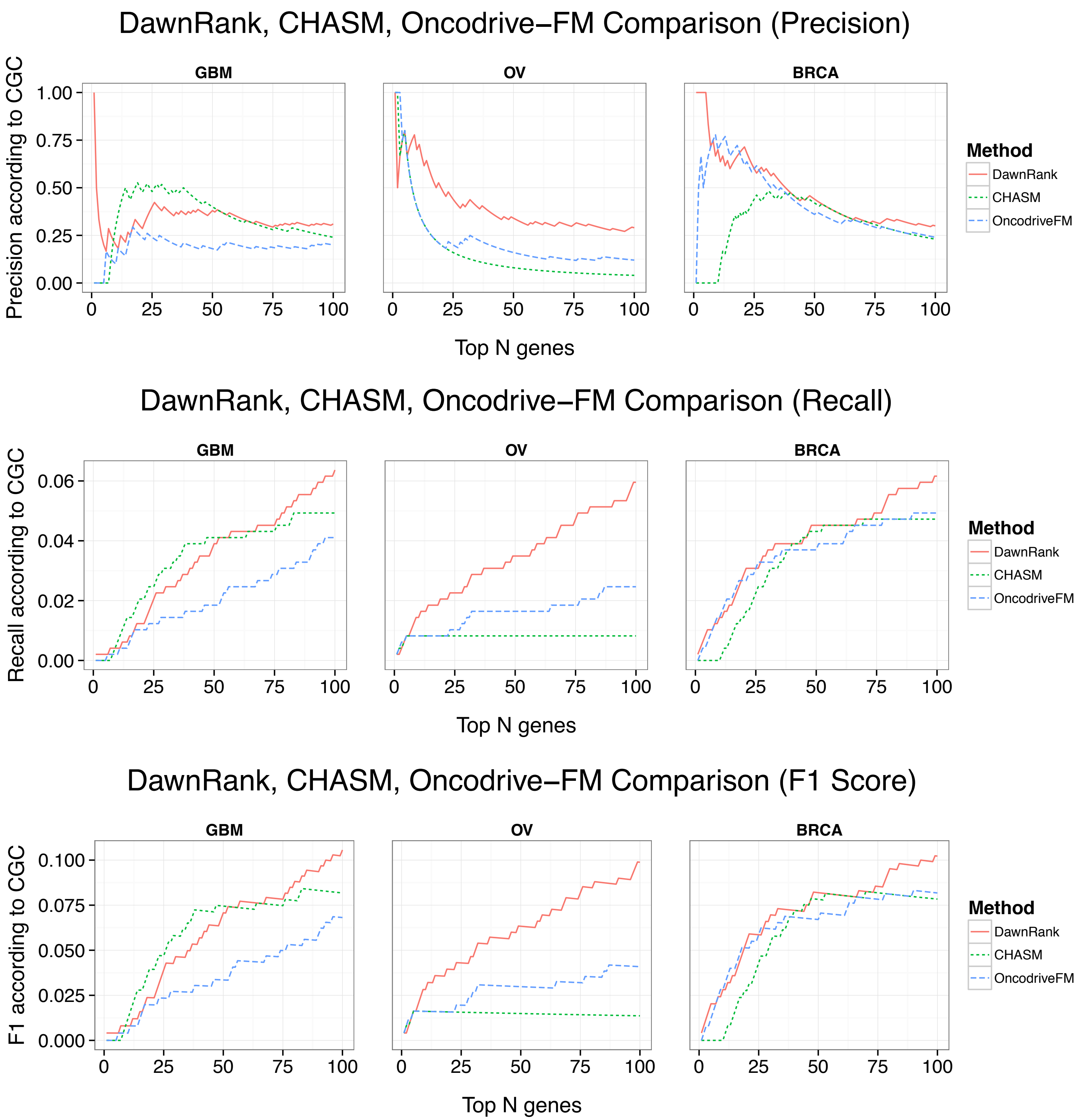

Figure S10: A comparison of the precision, recall, and F1-scores for the top ranking genes in DawnRank, CHASM and OncodriveFM. The X-axis represents the number of top ranking genes involved in the precision, recall, and F1 score calculation. The Y-axis represents the score of the given metric.

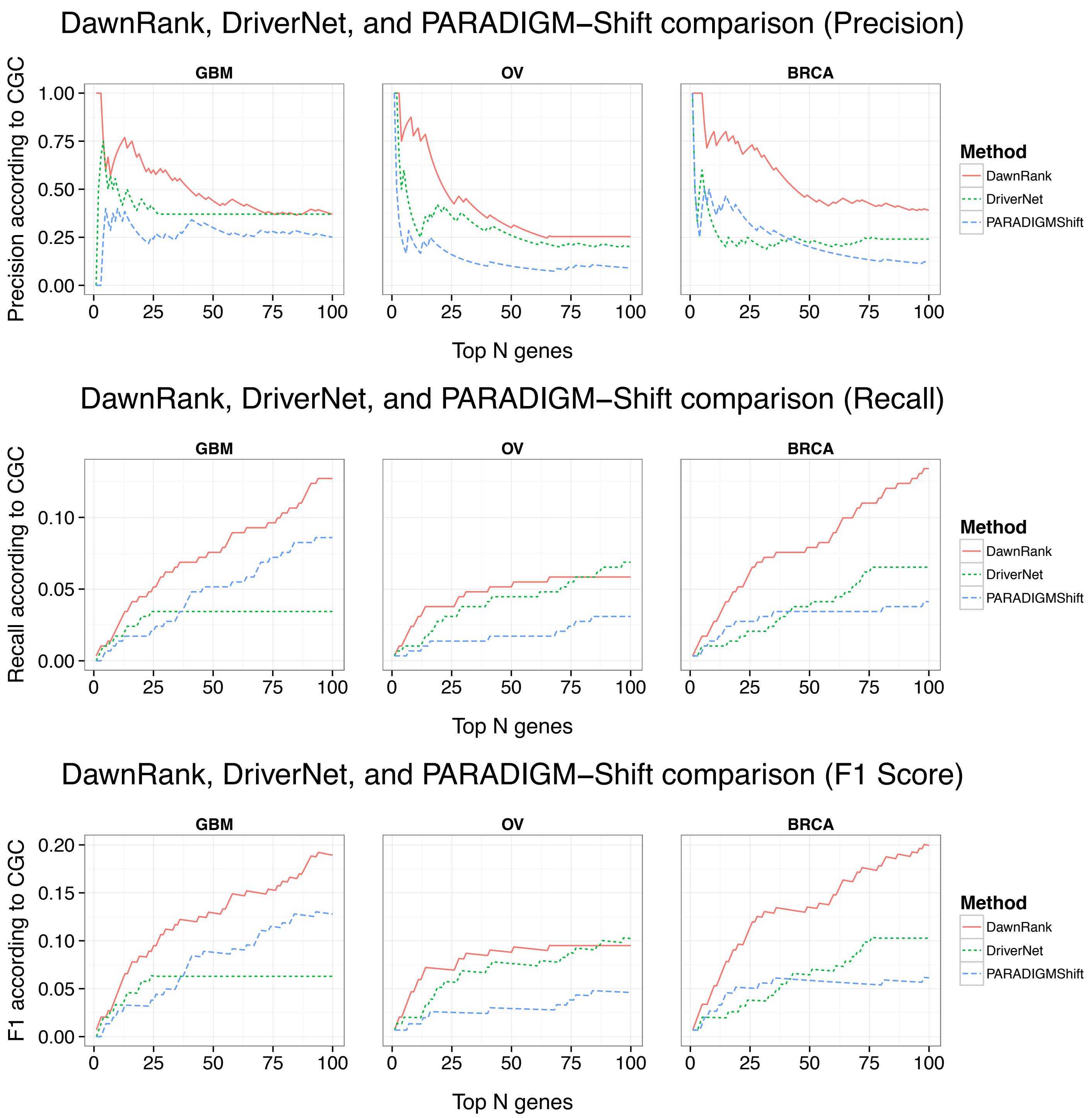

Figure S11: A comparison of the precision, recall, and F1-scores for the top ranking genes in DawnRank, DriverNet, and PARADIGM-Shift using the Pan-Cancer predicted drivers based on [4]. The X-axis represents the number of top ranking genes involved in the precision, recall, and F1 score calculation. The Y-axis represents the score of the given metric.

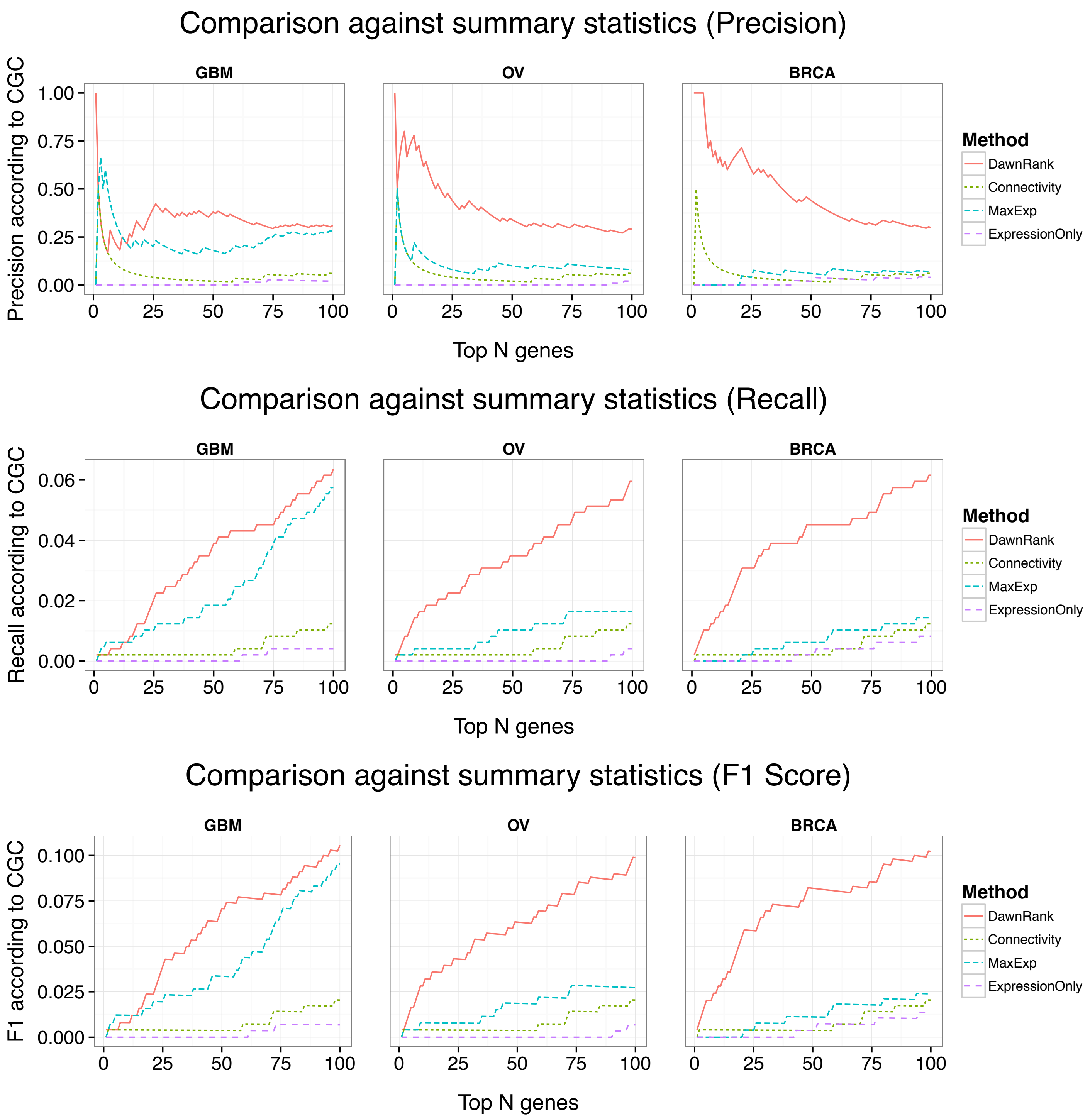

Figure S12: A comparison of the precision, recall, and F1-scores for the top ranking genes in DawnRank compared to three summary statistics: differential expression only, connectivity, and maximum downstream differential expression (MaxExp). The X-axis represents the number of top ranking genes involved in the precision, recall, and F1 score calculation. The Y-axis represents the score of the given metric.

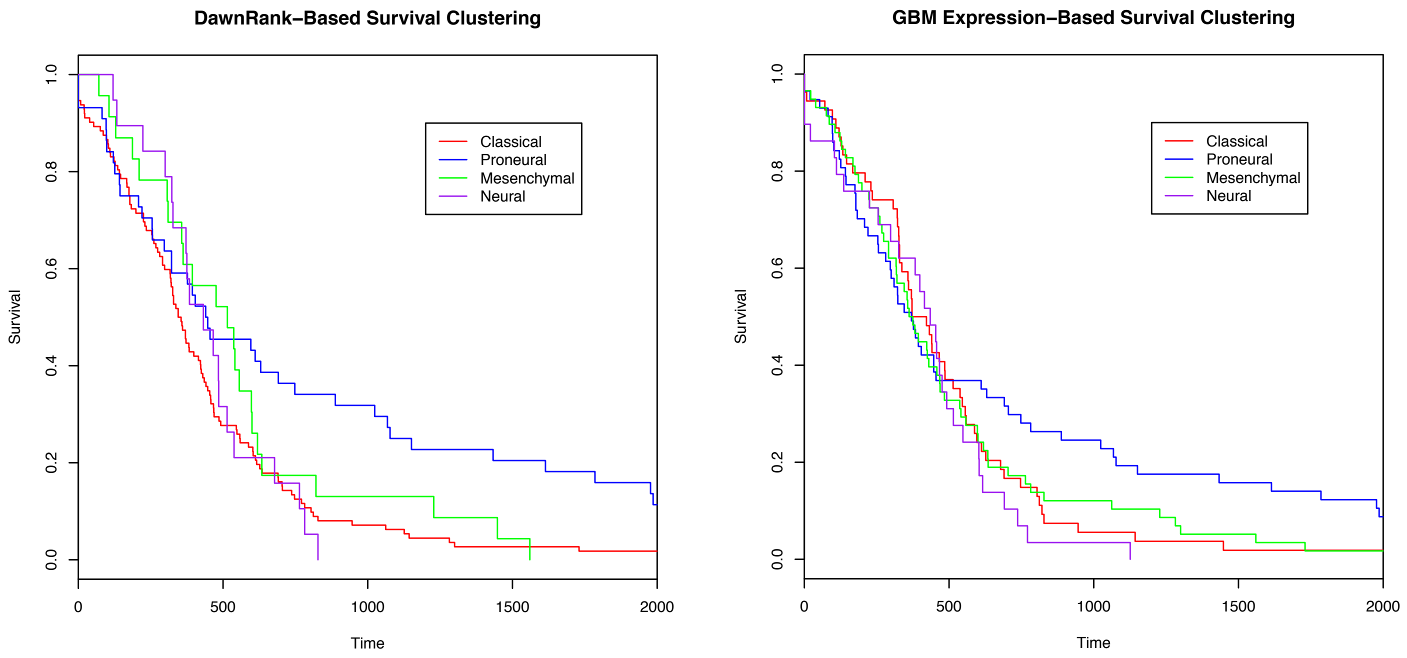

Figure S13: A Kaplan-Meier survival plot showing the survival rates of four clusters derived from using GBM DawnRank scores (left), as compared to expression based clustering (right). The X-axis represents the days until death and the Y-axis represents the proportion of living patients.

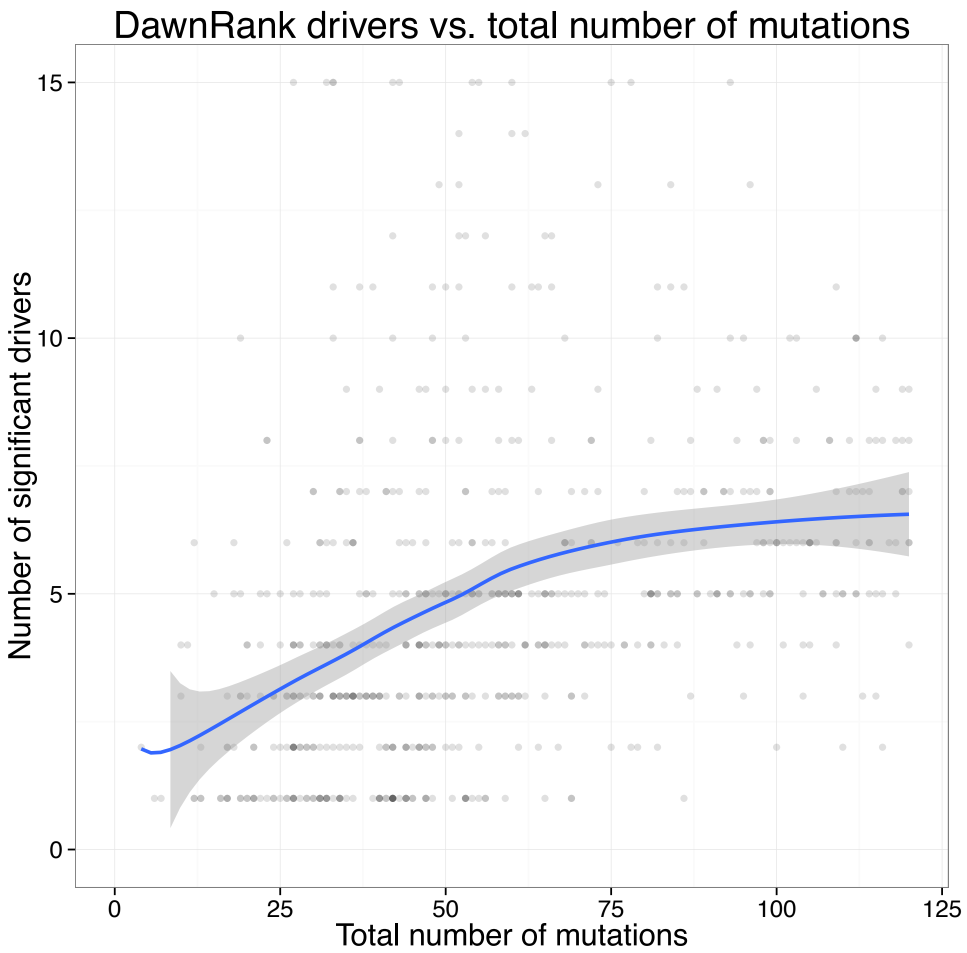

Figure S14: A plot illustrating the trend between the total number of mutations (X-axis) and the number of significant DawnRank drivers (Y-axis). The intensity of each point represents the number of patients at the coordinate. The line in blue is the LOESS curve.

References

1. Verhaak RG, Hoadley KA, Purdom E, Wang V, Qi Y, Wilkerson MD, Miller CR, Ding L, Golub T, Mesirov JP, et al: **Integrated genomic analysis identifies clinically relevant subtypes of glioblastoma characterized by abnormalities in PDGFRA, IDH1, EGFR, and NF1.** *Cancer Cell* 2010, **17:**98-110.
2. Marko NF, Weil RJ, Schroeder JL, Lang FF, Suki D, Sawaya RE: **Extent of resection of glioblastoma revisited: personalized survival modeling facilitates more accurate survival prediction and supports a maximum-safe-resection approach to surgery.** *J Clin Oncol* 2014, **32:**774-782.
3. Vogelstein B, Papadopoulos N, Velculescu VE, Zhou S, Diaz LA, Jr., Kinzler KW: **Cancer genome landscapes.** *Science* 2013, **339:**1546-1558.
4. Tamborero D, Gonzalez-Perez A, Perez-Llamas C, Deu-Pons J, Kandoth C, Reimand J, Lawrence MS, Getz G, Bader GD, Ding L, Lopez-Bigas N: **Comprehensive identification of mutational cancer driver genes across 12 tumor types.** *Sci Rep* 2013, **3:**2650.
